# Exploring common genomic biomarkers to disclose common drugs for the treatment of colorectal cancer and hepatocellular carcinoma with type-2 diabetes through transcriptomics analysis

**DOI:** 10.1371/journal.pone.0319028

**Published:** 2025-03-24

**Authors:** Sabkat Mahmud, Alvira Ajadee, Arnob Sarker, Reaz Ahmmed, Tasfia Noor, Md. Al Amin Pappu, Md. Saiful Islam, Md. Nurul Haque Mollah

**Affiliations:** 1 Bioinformatics Lab (Dry), Department of Statistics, University of Rajshahi, Bangladesh; 2 Department of Biochemistry & Molecular Biology, University of Rajshahi, Bangladesh; 3 Department of Computer Science and Engineering, Rajshahi University of Engineering & Technology (RUET), Bangladesh; The First Hospital of Jilin University, CHINA

## Abstract

Type 2 diabetes (T2D) is a crucial risk factor for both colorectal cancer (CRC) and hepatocellular carcinoma (HCC). However, so far, there was no study that has investigated common drugs against HCC and CRC during their co-occurrence with T2D patients. Consequently, patients often require multiple disease-specific multiple drugs, which can lead toxicities and adverse effects to the patients due to drug-drug interactions. This study aimed to identify common genomic biomarkers (cGBs) and associated pathogenetic mechanisms underlying CRC, HCC, and T2D to uncover potential common therapeutic compounds against these three diseases. Firstly, we identified 86 common differentially expressed genes (cDEGs) capable of separating each of CRC, HCC and T2D patients from control groups based on transcriptomic profiling. Of these cDEGs, 37 genes were upregulated and 49 were downregulated. Genetic association studies based on average of Log2 fold-change (aLog2FC) of cDEGs suggested a genetic association among CRC, HCC and T2D. Subsequently, six top-ranked cDEGs (MYC, MMP9, THBS1, IL6, CXCL1, and SPP1) were identified as common genomic biomarkers (cGBs) through protein-protein interaction (PPI) network analysis. Further analysis of these cGBs with GO-terms and KEGG pathways revealed shared pathogenetic mechanisms of three diseases, including specific biological processes, molecular functions, cellular components and signaling pathways. The gene co-regulatory network analysis identified two transcription factors (FOXC1 and GATA2) and three miRNAs (hsa-mir-195-5p, hsa-mir-124a-3p, and hsa-mir-34a-5p) as crucial transcriptional and post-transcriptional regulators of the cGBs. Finally, cGBs-guided seven candidate drugs (Digitoxin, Camptosar, AMG-900, Imatinib, Irinotecan, Midostaurin, and Linsitinib) as the common treatment against T2D, CRC and HCC were identified through molecular docking, cross-validation, and ADME/T (Absorption–Distribution–Metabolism–Excretion–Toxicity) analysis. Most of these findings received support by the literature review of diseases specific individual studies. Thus, this study offers valuable insights for researchers and clinicians to improve the diagnosis and treatment of CRC and/or HCC patients during the co-occurrence of T2D.

## 1. Introduction

Type 2 diabetes (T2D) is a chronic metabolic disease marked by impaired insulin secretion and function, and it accounts for over 90% of diabetes cases worldwide. Its prevalence has been rising globally, posing a significant health burden [1,2]. Key factors associated with T2D, including hyperinsulinemia and elevated levels of hepatokines such as insulin-like growth factor 1 (IGF-1), disrupt vital signaling pathways that regulate cell survival, stress responses, and apoptosis. These disruptions promote cancer cell growth and proliferation [3]. Population-based studies have consistently shown a strong association between T2D and increased risks of hepatocellular carcinoma (HCC) [4] and colorectal cancer (CRC) [5]. For instance, a U.S. cohort study of over 5 million patients revealed a 2- to 3-fold increased risk of HCC in individuals with T2D, particularly among those with long-standing or poorly managed diabetes [6]. Similarly, a Taiwanese study of over 480,000 individuals with T2D confirmed an elevated HCC risk, especially when combined with other metabolic conditions [7]. In the case of CRC, a meta-analysis of more than 2 million participants found a 27% higher risk among those with T2D [8], while a Swedish cohort study involving 400,000 diabetic patients reported a 30% higher CRC incidence, particularly in men [9].

**Fig 1:**
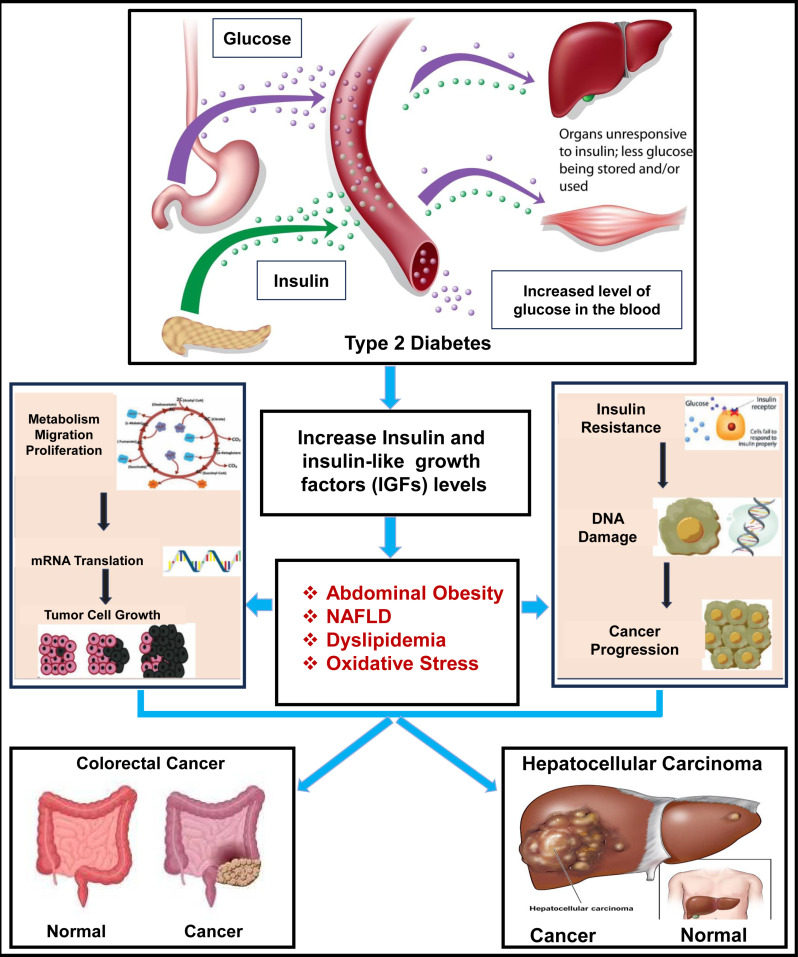
A schematic diagram about the link of T2D with HCC and CRC.

In individuals with T2D, factors like abdominal obesity, NAFLD, dyslipidemia, oxidative stress, etc. significantly drive the development of HCC and CRC [10]. Abdominal obesity often leads to chronic inflammation and insulin resistance, creating a metabolic environment that fosters cancer progression [11]. NAFLD raises the risk of HCC by driving liver inflammation and fibrosis, while its links to insulin resistance and systemic inflammation promote CRC development [12,13]. Dyslipidemia, characterized by abnormal lipid levels, contributes to cellular damage in the liver and colon by promoting lipid peroxidation and inflammation, both of which support tumor growth [14]. Oxidative stress further exacerbates these effects by causing DNA damage, impairing cellular repair mechanisms, and enhancing cellular proliferation, collectively increasing the likelihood of malignant transformation in liver and colon tissues [15]. These interlinked metabolic disturbances create a pro-carcinogenic environment, raising the risk of both HCC and CRC. A schematic diagram about the connection of T2D with CRC and HCC has been given in Fig 1.

Doctors typically prescribe drugs that are specific to the patient’s disease [16]. Therefore, when treating patients with multiple diseases, doctors often need to prescribe diseases specific several medications. However, polypharmacy can lead to drug-drug interactions (DDIs), which may cause adverse effects or toxicity, potentially worsening the patient’s health [17–20]. In such situations, doctors should aim to prescribe a smaller number of common drugs that effectively represent the specific medications of those diseases, thereby reducing toxicity. However, so far, there is currently no study in the literature that has suggested a common drug for treating CRC and/or HCC patients who also have T2D, despite older patients being at a higher risk of drug-drug interactions (DDIs) due to polypharmacy and age-related metabolic changes. Therefore, the main aim of this study is to identify common genomic biomarkers (cGBs) and associated pathogenetic mechanisms underlying CRC, HCC, and T2D to uncover potential common therapeutic compounds against these three diseases.

## 2. Methodology

To identify common drugs for T2D, HCC, and CRC, we first need to explore common genomic biomarkers (cGBs) linked to all three conditions. Transcriptomics profile analysis through bioinformatics tools is a popular approach to detect disease-causing key genes (GBs) as drug-targets [21–26].

### 2.1 Data sources and descriptions

#### 2.1.1 Collection of transcriptomics profiles (case/control).

Several studies in the literature have investigated common genomic biomarkers (cGBs) associated with multiple diseases using independent transcriptomics datasets [27–30]. In our study, we aimed to identify cGBs linked to CRC, HCC, and T2D by analyzing datasets from the NCBI database: GSE9348 for CRC, GSE121248 for HCC, and GSE76896 for T2D. All datasets were generated on the Affymetrix GPL570 platform. We began by downloading the CEL files for each dataset. To ensure accurate data processing, we used the Affy package and applied the Gene-Chip Robust Multichip Average (GC-RMA) algorithm [31] with the hgu133plus2.db chip [32] for background correction and normalization of all microarray files.

#### 2.1.2 Collection of candidate drug molecules.

The development of new drugs is inherently complex, time-consuming, and expensive. In contrast, repurposing existing drugs offers a cost-effective and time-saving approach to treatment development, as these drugs already have established safety profiles [33,34]. Repurposed drugs can often enter clinical trials more quickly, potentially providing faster therapeutic options for patients [35]. This strategy is especially beneficial for managing complex conditions such as T2D, CRC, and HCC, as it allows for shared treatments that can help minimize the adverse effects associated with multiple medications [36].

To explore repurposable drugs for the shared treatment of T2D, HCC, and CRC, we collected a total of 106 drugs for CRC (S1 Table), 126 drugs for HCC (S2 Table) and 237 drugs for T2D (S3 Table) to explore repurposable common drugs for the treatment of CRC and/or HCC with T2D. Additionally, we collected key genomic biomarkers for each condition—CRC (S4), HCC (S5 Table), and T2D (S6 Table)—and considered those biomarkers that are repeated in multiple studies to validate proposed drugs.

### 2.2 Identification of differentially expressed genes (DEGs) between case and control groups

At first differentially expressed genes (DEGs) between cases (T2D/CRC/HCC) and control groups were identified by using “CEMiTool” [37] from three datasets: GSE9348 for CRC, GSE121248 for HCC, and GSE76896 for T2D. The detail discussion can be found in our previous study about how CEMiTool provides DEGs between disease and control groups [38]. Then weighted gene co-expression network analysis (WGCNA) [39] technique was used for further filtering of those DEGs by removing the clusters (modules) of less correlated DEGs. Module-trait relationships were determined by computing the Pearson correlation between module eigengenes (MEs) and traits. Modules with significant correlations (|*r* | ≥  0.6, *p*-value < 0.001) were selected for further analysis. DEGs were identified by combining all genes from significant modules for each condition (T2D, CRC, HCC). We then separated upregulated (UR) and downregulated (DR) DEGs using the criteria of aLog_2_FC_i_ >  1 and aLog_2_FC_i_ < -1, respectively, where aLog_2_FC values indicate the average of Log_2_ fold-change values and are computed as


$$aLog_{2}FC_{g}= \{\begin{array}{c} \frac{1}{n_{1}}\overset{n_{1}}{\underset{i}{\sum }}log_{2}(z_{gi}^{D})-\frac{1}{n_{2}}\overset{n_{2}}{\underset{j}{\sum }}Log_{2}(z_{gj}^{C}),ifn_{1}\neq n_{2}\\ \frac{1}{n}\overset{n}{\underset{i}{\sum }}log_{2}\left(\frac{z_{gi}^{D}}{z_{gj}^{C}}\right),ifn_{1}=n_{2}=n \end{array}$$ (1)

Here $z_{gi}^{D}$ and $z_{gj}^{C}$ represent the responses of the *gt*h gene in the *i*th disease sample and *j*th normal sample, respectively.

### 2.3 Identification of common DEGs (cDEGs) among T2D, CRC and HCC

According to the previous discussion in section 2.2, the upregulated (UR) and downregulated (DR) DEGs between CRC and control samples were computed from the dataset with NCBI accession-ID GSE9348, the UR- and DR-DEGs between HCC and control groups from the dataset with accession-ID GSE121248, and the UR- and DR-DEGs between T2D and control groups from the dataset with accession-ID GSE76896. Then, common UR- and DR-DEGs for T2D, CRC and HCC were combined to create a unified set of common DEGs (cDEGs) as follows.


$$\text{cDEGs}={\text{cDEGs}}^{\text{UR}}\cup {\text{cDEGs}}^{\text{DR}}$$ (2)

where,


$${\text{cDEGs}}^{\text{UR}}={\text{DEGs}}_{\text{T}2\text{D}}^{\text{UR}}\cap {\text{DEGs}}_{\text{CRC }}^{\text{UR}}\cap {\text{DEGs}}_{\text{HCC}}^{\text{UR}}$$

and


$${\text{cDEGs}}^{\text{DR}}={\text{DEGs}}_{\text{T}2\text{D}}^{\text{DR}}\cap {\text{DEGs}}_{\text{CRC}}^{\text{DR}}\;\cap {\text{DEGs}}_{\text{HCC}}^{\text{DR}}$$

Here ${\text{DEGs}}_{\text{T}2\text{D}}^{\text{UR}},{\text{DEGs}}_{\text{HCC}}^{\text{UR}},\text{and}{\text{DEGs}}_{\text{CRC}}^{\text{UR}}$ indicates the upregulated (UR) DEGs for T2D, HCC and CRC, respectively. On the other hand, ${\text{DEGs}}_{\text{T}2\text{D}}^{\text{DR}},{\text{DEGs}}_{\text{HCC}}^{\text{UR}},\text{and}{\text{DEGs}}_{\text{CRC}}^{\text{DR}}$ represents the downregulated (DR) DEGs for T2D, HCC, and CRC, respectively. The symbols ‘∪’ and ‘∩’ indicate the union and intersection of set operation, respectively. Thus, cDEGs as well as both ${\text{cDEGs}}^{\text{UR}}$ and ${\text{cDEGs}}^{\text{DR}}$ can separate each of T2D, CRC and HCC samples from the control samples (S5 Fig).

### 2.4 Investigating local genetic association of CRC and HCC with T2D through cDEGs

According to the previous discussion in sections 2.2 and 2.3, CRC, HCC and T2D might be locally and positively associated with each other due to their similar trends of aLog2FC values across cDEGs. To quantify this local genetic association, simple correlation coefficient (*r*) is employed as follows


$$r_{ab}=\frac{\sum \left(a_{i}-\bar{a}\right)\left(b_{i}-\bar{b}\right)}{\sqrt{\sum {\left(a_{i}-\bar{a}\right)}^{2}{\left(b_{i}-\bar{b}\right)}^{2}}}$$ (3)

where, $a_{i}=\text{a}Log_{2}{\text{FC}}_{i}\left(A\right)$ and $b_{i}=\text{a}Log_{2}FC_{i}\left(B\right)$ are the aLog_*2*_FC values of the *i*^th^ gene for the two diseases *A* and *B*, respectively; $\bar{a}$ and $\bar{b}$ are the means of ai's and bi's, respectively. This measure was also used in a previous study to investigate the local genetic association between two diseases [40].

### 2.5 Identification of three disease-causing common genomic biomarkers (cGBs)

In this study, top-ranked cDEGs among T2D, CRC and HCC were considered as the common genomic biomarkers (cGBs). In order to explore top-ranked cDEGs, the STRING v11.5 database and analysis tool were used to construct a protein-protein interaction (PPI) network for the common differentially expressed genes (cDEGs). To generate a PPI network, the distance matrix D is calculated using,


$$\text{D}\left(\text{a},\text{b}\right)=\frac{2\left|N_{a}\cap N_{b}\right|}{|N_{a}\left|+|N_{b}\right|}$$

where *N*_*a*_ represents the set of neighboring proteins for the *a*th protein and *N*_*b*_ represents the set of neighboring proteins for the *b*th protein. The PPI network was visualized using Cytoscape software [41]. To identify the top-ranked cDEGs as cGBs, the CytoHubba plugin in Cytoscape [42] was employed, using six topological measures: Closeness, Degree, Edge Percolated Component (EPC), Maximal Clique Centrality (MCC), Maximum Neighborhood Component (MNC), and Density of Maximum Neighborhood Component (DMNC). Additionally, the Molecular Complex Detection (MCODE) analysis was utilized to detect the most significant modules of PPI network [43]. Obviously, cGBs can separate each of T2D, CRC and HCC samples from the control samples.

### 2.6 Verification on the association of cGBs with T2D, CRC and HCC through independent databases

To verify associations between common gene biomarkers (cGBs) and diseases like T2D, CRC, and HCC, we used the Enrichr tool [44], specifically analyzing the DisGeNET database [45] for disease-cGB enrichment. DisGeNET integrates comprehensive gene-disease associations, which enables robust exploration of genetic links to diseases. Using Enrichr’s statistical framework, we calculated *p*-values for enrichment with the Fisher Exact Test to estimate the probability that observed overlaps between cGBs and disease genes could occur by chance. These *p*-values were then corrected using the Benjamini-Hochberg method to control the false discovery rate (FDR), reducing Type I errors. Enrichr’s combined score, which factors in both *p*-value and z-score, provides an adjusted measure of association strength, where lower *p*-values indicate significant associations, affirming the potential relevance of cGBs to each disease.

### 2.7 Verification of differential expression patterns of cGBs with T2D, CRC and HCC based on independent datasets

The expression patterns of cGBs were independently validated for T2D, CRC, and HCC using box plot analysis with datasets from the NCBI database. The variation in expression levels of cGBs between CRC, HCC, and control samples was validated using data from the TCGA and GTEx databases via the GEPIA2 web tool [46]. Independent datasets were utilized to validate the differential expression patterns of cGBs between T2D and control samples, and box plots were constructed to confirm these patterns among T2D, CRC, HCC, and their respective control groups. To further evaluate the predictive capability of these cGBs, we constructed a Random Forest (RF) model using three independent expression profiles from the NCBI database. This model was assessed by generating Receiver Operating Characteristic (ROC) curves, employing the R-package “ROCR” [47] to calculate the Area Under the Curve (AUC) values. AUC serves as a quantitative measure of model performance, where higher AUC values indicate stronger predictive accuracy of cGBs for distinguishing between disease and control groups. This approach provides robust statistical validation of cGB expression patterns and predictive relevance across T2D, CRC, and HCC.

### 2.8 Revealing common pathogenetic processes of T2D, CRC and HCC with cGBs

We conducted enrichment analysis using Gene Ontology (GO) terms and KEGG pathways to examine the shared pathogenetic mechanisms underlying T2D, CRC, and HCC through cGBs. This analysis helped identify the key biological processes and pathways associated with the cGBs. Additionally, we performed regulatory network analysis to investigate the roles of transcription factors (TFs) and microRNAs in regulating these biomarkers. The methods for these analyses are described in detail in subsections 2.8.1 and 2.8.2.

#### 2.8.1 Regulatory network analysis of cGBs.

We performed regulatory network analysis with transcription factors (TFs) and microRNAs (miRNA) to explore the key regulators of cGBs. In order to determine the primary TFs connected with cGBs, we analyzed the TFs-KGs connection network with the JASPAR database [48]. By examining the links between miRNA and cGBs using the TarBase [49] databases, it was possible to identify the significant miRNAs that have an impact on cGBs at the post-transcriptional stage. NetworkAnalyst [50] was used to replicate these interactions. The post-transcriptional regulators of cGBs were selected from top-ranked miRNAs. We used Cytoscape [51] to visualize the networks of their interactions.

#### 2.8.2 The cGBs-set enrichment analysis with GO-terms and KEGG-pathways.

The Gene Ontology (GO) project is a bioinformatics tool that uses domain-specific ontologies to provide a complete source of functional data on gene products and descriptions of activities [52]. To investigate the gene ontology and KEGG pathway of cGBs, we considered the DAVID web server [53], with an *p*-value of 0.05 chosen as the threshold, which is determined by Fisher’s exact test. To identify significantly enriched GO terms (biological processes, molecular functions, cellular components) and KEGG pathways associated with the set of significant cGBs, a 2 × 2 contingency table was created (Table 1).

**Table 1:** A  2 × 2 contingency table.

Annotated genes	cGBs (proposed)	Not- cGBs	Marginal total
Annotated gene-set in *x*^th^ GO term/KEGG pathway (*A*_*x*_)	*k*_*x*_	*M*_*x*_ *- k*_*x*_	*M*_*x*_
Complement gene-set of *A*^*c*^_(_$A_{x}^{c}$)	*n - k*_*x*_	*N - M*_*x*_ *– n* + *kx*	*N - M*_*x*_
Marginal total	*n*	*N - n*	*N* (Grand total)

### 2.9 The cGBs-guided drug repurposing

To investigate potential repurposable drug molecules guided by cGBs for T2D, HCC, and CRC, we conducted a comprehensive analysis that included molecular docking and an evaluation of ADME/T (Absorption, Distribution, Metabolism, Excretion, and Toxicity) properties. Molecular docking was used to calculate binding affinity scores and interaction patterns between the identified cGBs and drug candidates, allowing us to assess their therapeutic potential. Following this, we performed ADME/T analysis on the top-ranked drug molecules to evaluate their pharmacokinetic and toxicity profiles, ensuring their suitability for further drug development and clinical application. Together, these methodologies were employed to identify promising drug candidates based on their interactions with cGBs and to assess their pharmacokinetic properties, as detailed in subsections 2.9.1 and 2.9.2.

#### 2.9.1 Molecular docking.

To investigate potential drug repurposing opportunities, we conducted molecular docking between cGB-mediated receptor proteins and repurposable drug molecules using AutoDock Vina [54]. The cGBs and their associated top TF proteins were considered as target receptors. A total of 469 candidate drug molecules, collected from published studies and databases related to T2D, HCC, and CRC, were used for docking, as detailed in S1, S2, and S3 Table. The 3D structures of the receptor proteins were sourced from the Protein Data Bank [55] and AlphaFold databases [56], while the 3D structures of the candidate drugs were obtained from the PubChem database [57]. Molecular docking was performed to compute the binding affinity scores (in kcal/mol) between each receptor and drug molecule. To identify the top-ranked drug candidates, receptor proteins were arranged in descending order of average binding affinity score, and drug molecules were similarly ranked by decreasing column averages in the resulting score matrix.

#### 2.9.2 ADME/T analysis.

ADME/T analysis examines a drug candidate’s absorption, distribution, metabolism, excretion, and toxicity to assess its safety and therapeutic potential. In drug repurposing, while a compound may exhibit high binding affinity to a novel target in docking studies, its success heavily depends on meeting ADMET criteria. Poor absorption, rapid metabolism, or high toxicity can render a promising candidate unsuitable for clinical use. Therefore, ADMET evaluation is crucial for identifying compounds with favorable pharmacokinetic and safety profiles, ensuring only viable candidates proceed in the repurposing pipeline. To assess the pharmacokinetic properties of selected compounds, ADMET analysis was conducted. The SCFBio platform was utilized to determine drug-likeness based on the Lipinski rule of five, which considers parameters such as molecular weight, hydrogen bond donors and acceptors, rotatable bond count, and the LogP (partition coefficient). Additionally, SwissADME [58] and pkCSM [59] tools were employed to predict ADMET characteristics, using the SMILES representation of the compounds to evaluate their structural and pharmacokinetic properties.

### 2.10 DFT analysis

Density Functional Theory (DFT) is a quantum mechanical method used to model the electronic structures of molecules, offering valuable insights into their properties and interactions. In the context of drug discovery, DFT aids in predicting binding affinities, optimizing lead compounds, and assessing the stability and toxicity of potential drugs, thus facilitating the development of effective and safe therapeutic agents [60–63]. In this study, we performed DFT calculations on the seven proposed drug molecules using Gaussian 09 software [64]. We calculated the energy of frontier molecular orbitals (FMO), including the Highest Occupied Molecular Orbital (HOMO) and the Lowest Unoccupied Molecular Orbital (LUMO), along with other quantum chemical descriptors. These calculations were conducted in the ground state of the compounds using the B3LYP method and the 6-311G basis set through the GaussView-6 interface. The following equations were employed to compute various quantum chemical parameters [65]


$$\text{Energy gap }\Delta \text{E}={\text{E}}_{\text{LUMO}}-{\text{E}}_{\text{HOMO}}$$ (4)

$$\text{Ionization Potential I}=-{\text{E}}_{\text{HOMO}}$$ (5)

$$\text{Electron Affinity A}=-\;{\text{E}}_{\text{LUMO}}$$ (6)

$$\text{Chemical Hardness}\eta =\frac{I-A}{2}$$ (7)

$$\text{Chemical Softness}\sigma =\frac{1}{n}$$ (8)

$$\text{Electronegativity χ}=\frac{I+A}{2}$$ (9)

Chemical Potentialµ=−χ (10)

### 2.11 Molecular dynamics simulation

Molecular Dynamics (MD) simulations were employed to validate molecular docking results and assess the stability of protein-ligand complexes [66]. Simulations were conducted for 100 ns using Gromacs 2020 and the CHARMM36 force field [67,68]. The system was solvated in a periodic orthorhombic box filled with TIP3P water, 10 Å buffer spacing, and neutralized with 0.15 M NaCl. Energy minimization was performed using the steepest descent method, and the system was equilibrated at 310 K under NVT and NPT ensembles [69].

The electrostatic energy was calculated using the Particle Mesh Ewald (PME) method:


$$E_{electrostatic}=\overset{N}{\underset{i=1}{\sum }}\underset{j\neq i}{\sum }\frac{q_{i}q_{j}}{r_{ij}}+\text{reciprocal lattice contributions}$$

Where, *N* Total number of particles in the system, $q_{i}\text{and }q_{j}$ are the charges of particles *i* and *j*, $r_{ij}$ Distance between particles *i* and *j*.

The stability of the protein-ligand complexes was assessed through Root Mean Square Deviation (RMSD) and Root Mean Square Fluctuation (RMSF):


$$\text{RMSD}=\sqrt{\frac{\sum_{i=1}^{N}{\left(\tau_{i}-\tau_{0}\right)}^{2}}{N}}$$

*N* is the number of atoms or residues in the protein-ligand complex, $\tau_{i}$ is the position of atom *i* at a specific time frame and $\tau_{0}is$ reference position of atom *i*, typically from the initial frame.


$$\text{RMSF}=\sqrt{\frac{\sum_{i=1}^{T}{\left(\tau_{i,t}-{\bar{\tau }}_{i}\right)}^{2}}{T}}$$

Where $\tau_{i,t}$ are the position of atom *i* at time *t*, respectively and T is the total number of time frames in the trajectory.

The binding free energy (ΔGbind) of the protein-ligand interactions was calculated for each snapshot using the MM-GBSA method, implemented via the gmx_MM-PBSA tool. The binding free energy was determined using the equation:


$$\Delta G_{bind}=E_{complex}–\left(E_{protein}+E_{ligand}\right)$$

This comprehensive approach considered multiple components of interaction, including van der Waals forces, electrostatic interactions, polar solvation effects, solvent-accessible surface area (SASA) contributions, and overall binding energies. Trajectories from molecular dynamics (MD) simulations performed in explicit water (using the Desmond module) were converted to GROMACS-compatible formats using InterMol software [70–72].

## 3. Results

This section summarizes the comprehensive findings, including the discovery of cGBs, their functional roles in T2D, CRC, and HCC, and the discovery of potential therapeutic drug candidates through integrative bioinformatics analyses.

### 3.1 DEGs Identification

Differentially expressed genes (DEGs) for T2D, CRC, and HCC were identified using the “CEMiTool” and “WGCNA” approaches across three datasets: GSE9348, GSE121248, and GSE76896. Initially, the “CEMiTool” analysis identified 4,589 DEGs from GSE9348, 4,588 DEGs from GSE121248, and 7,548 DEGs from GSE76896. These DEGs were further refined using the “WGCNA” approach. Outlier detection based on a cut height threshold >  250 confirmed no outliers in the datasets (S1 Fig). For network construction, we selected optimal soft-thresholding power (β) values for each dataset: β =  14 for GSE76896, β =  11 for GSE9348, and β =  15 for GSE121248. The selection was based on achieving a scale-free topology with a cutoff R² value of 0.84 (S2 Fig). Using the selected β values, adjacency matrices were constructed, followed by hierarchical clustering using the topological overlap matrix (TOM) dissimilarity measure. Modules were defined with a minimum size of 30 genes and merged with a module eigengene cut height of 0.17 (S3 Fig). Module-trait relationships were evaluated, selecting modules with correlation values >  0.6 or < -0.6 and *p-*values < 4e-71. This analysis resulted in the identification of five modules for GSE76896 and three modules each for GSE9348 and GSE121248 (S4 Fig). After filtering through the “WGCNA” approach, the final number of DEGs for each dataset was determined. GSE76896 revealed 2,327 DEGs, including 1,435 upregulated and 892 downregulated genes. GSE9348 identified 3,125 DEGs, comprising 1,919 upregulated and 1,206 downregulated genes, while GSE121248 uncovered 2,402 DEGs, with 1,266 upregulated and 1,136 downregulated genes. This combined approach ensured the identification of biologically significant DEGs across the datasets.

### 3.2 Identification of common DEGs (cDEGs)

**Fig 2:**
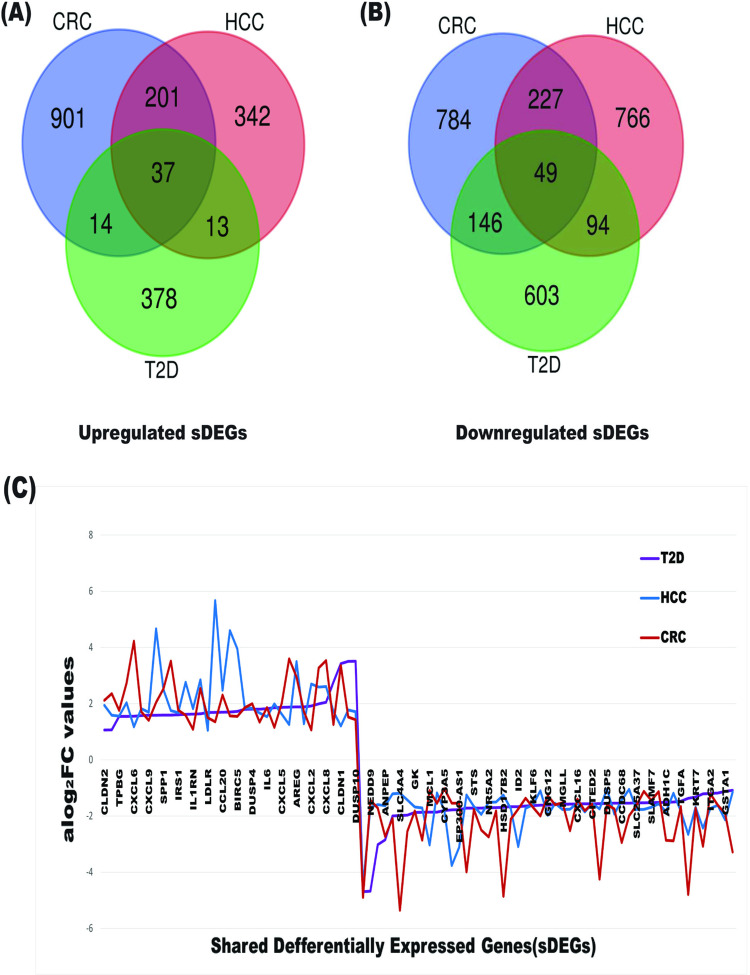
Venn diagrams for DEGs and the line charts for the trends of aLog_2_FC values with common DEGs (cDEGs) in T2D, HCC, and CRC. (A) Venn diagrams for upregulated DEGs; (B) Venn diagrams for downregulated DEGs; and (C) line charts for the trends of aLog_2_FC values with cDEGs in T2D, HCC, and CRC.

We identified a total of 86 common differentially expressed genes (cDEGs) shared across all three comparisons: control vs. CRC, control vs. T2D, and control vs. HCC. Among these cDEGs, 37 genes were upregulated, while 49 were downregulated. The complete list of these cDEGs is provided in S7 Table. To illustrate these findings, a Venn diagram highlights the overlap of cDEGs across the comparisons (Fig 2A and 2B), while their expression trends are depicted in a line chart (Fig 2C). Additionally, a heatmap presenting the expression profiles of these cDEGs for each disease type is shown in S5 Fig. These visualizations provide a comprehensive overview of the shared genetic alterations associated with CRC, T2D, and HCC.

### 3.3 Genetic association among T2D, HCC and CRC through cDEGs

To understand the link of T2D with CRC and HCC, we computed pairwise local correlation coefficients using equation 2 for T2D, HCC and CRC based on the aLog_2_FC values of cDEGs (S8 Table). The correlation coefficient between each pair of the three diseases (T2D, HCC, and CRC) was ≥  0.82 (Table 2), suggesting that these conditions are locally interconnected through the expression of cDEGs.

**Table 2:** Local Correlation Matrix for T2D, HCC and CRC through cDEGs.

	T2D	HCC	CRC
T2D	1	0.85693	0.86124
HCC	0.85693	1	0.82001
CRC	0.86124	0.82001	1

### 3.4 Identification of common genomic biomarkers (cGBs)

**Fig 3:**
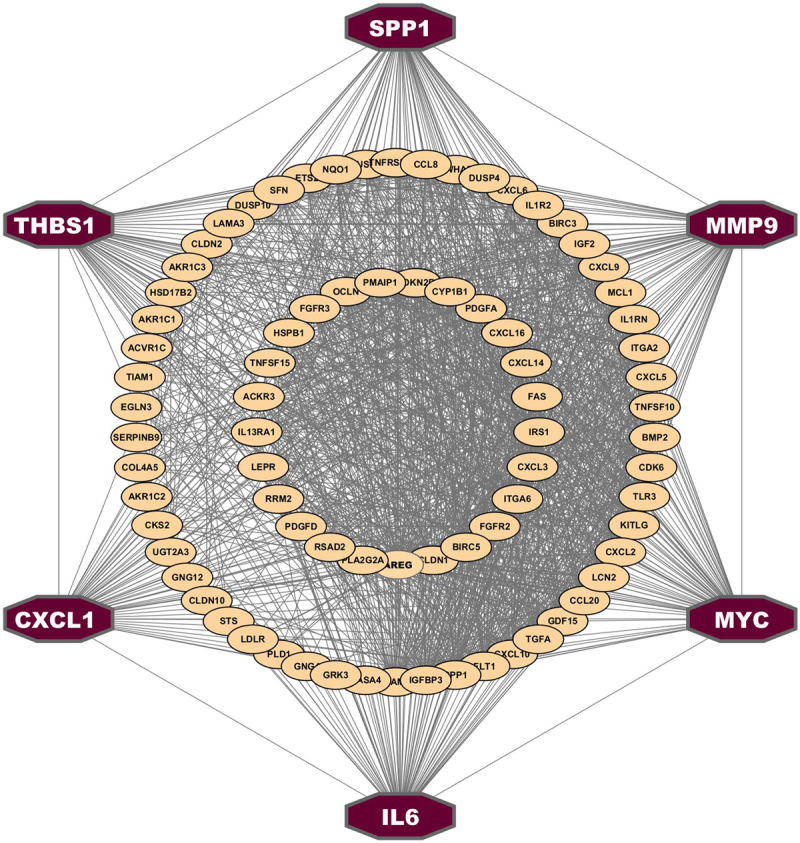
Protein-protein interactions (PPIs) network analysis of cDEGs. Octagon shape nodes with dark-red color indicate the cGBs.

The PPI network of cDEGs was constructed, comprising 86 nodes and 459 edges (Fig 3). We selected the top six cGBs (MYC, MMP9, SPP1, IL6, THBS1, and CXCL1) based on seven topological methods with the following thresholds: degree =  47, closeness =  64.833, EPC =  18.124, MNC =  46, betweenness =  179.307, radialty =  4.583, and stress =  1980 (S9 Table). Notably, all selected cGBs are upregulated.

### 3.5 Verification on the association of cGBs with T2D, CRC and HCC through independent databases

The disease- cGBs interaction analysis with ‘DisGenet-database’ showed that top-ranked 25 diseases (including T2D, HCC and CRC) are significantly (adjusted *p*-value < 0.05) associated with cGBs (S10 Table). The findings indicate that cGBs play a significant role in the pathogenesis of these diseases, highlighting their potential as therapeutic targets.

### 3.6 Verification of differential expression patterns of cGBs with T2D, CRC and HCC based on independent datasets and databases

We investigated the differential expression patterns of cGBs using two independent databases, GTEx and TCGA, which collectively included 349 case and 275 control samples for CRC and 160 case and 369 control samples for HCC, analyzed through box plots (S6A Fig). Additionally, we assessed the expression patterns of cGBs in T2D using the independent gene expression profile GSE15932 from the NCBI database, comprising 8 T2D and 8 control samples (S6B Fig). Our analysis revealed that all cGBs are upregulated across these three diseases, corroborating our earlier findings. To further evaluate the predictive accuracy of cGBs, we developed a Random Forest (RF) classification model, employing a 5-fold cross-validation approach to ensure robustness and reliability. We allocated 60% of the samples for training and used the remaining 40% as test data. We also included three additional independent test datasets (GSE8671 for CRC, GSE45267 for HCC, and GSE19420 for T2D) from the NCBI database. For each test dataset, we constructed ROC curves (S7 Fig) and calculated performance metrics (AUC, TPR, TNR, and accuracy) (S11 Table). The results demonstrated strong predictive performance for cGBs, with an AUC exceeding 0.9 and accuracy greater than 0.82 across the models.

### 3.7 Revealing common pathogenetic processes of T2D, CRC and HCC with cGBs

To uncover the common pathogenetic processes associated with T2D, CRC, and HCC through the cGBs, we conducted enrichment analysis using gene ontology (GO) terms and KEGG pathways. Additionally, we performed regulatory network analysis involving transcription factors (TFs) and microRNAs. The details of these analyses are discussed in the following subsections (3.7.1-3.7.2).

#### 3.7.1 The cGBs-set enrichment analysis with GO-terms and KEGG-pathways.

We conducted GO and KEGG pathway enrichment analyses for six cGBs to investigate the shared pathogenetic processes underlying T2D, CRC, and HCC, utilizing the DAVID web tool. Table 3 presents the top five enriched biological processes (BPs), molecular functions (MFs), cellular components (CCs), and KEGG pathways identified in this analysis.

**Table 3:** The top five significantly (*p-*value< < 0.01) enriched GO terms and KEGG pathways with cGBs by Enrichr.

Biological Process			
GO ID	GO term	*p*-value	Annotated cGBs
GO:0030330	chemokine-mediated signaling pathway	1.38 $\times 10^{-11}$	*CXCL1*
GO:0071222	cellular response to lipopolysaccharide	7.06 $\times 10^{-9}$	*IL6, CXCL1, MMP9*
GO:0006954	inflammatory response	1.67 $\times 10^{-7}$	*IL6, CXCL1, THBS1*
GO:0007165	signal transduction	1.42 $\times 10^{-5}$	*CXCL1, SPP1*
GO:0008284	positive regulation of cell proliferation	0.001499	*IL6, MYC, THBS1*
**Molecular Function**			
**GO ID**	**GO term**	*p* **-value**	**Associated cGBs**
GO:0045236	CXCR chemokine receptor binding	1.53 $\times 10^{-13}$	*CXCL1*
GO:0008009	chemokine activity	1.03 $\times 10^{-12}$	*CXCL1*
GO:0005125	cytokine activity	2.07 $\times 10^{-4}$	*IL6, SPP1*
GO:0005515	protein binding	9.19 $\times 10^{-4}$	*CXCL1, MMP9, MYC*
GO:0042802	identical protein binding	0.001258	*MYC, MMP9*
**Cellular Component**			
**GO ID**	**GO term**	*p-* **value**	**Associated cGBs**
GO:0005615	extracellular space	1.73 $\times 10^{-10}$	*CXCL1, MMP9, SPP1, IL6, THBS1*
GO:0005576	extracellular region	1.10 $\times 10^{-5}$	*CXCL1, MMP9, SPP1, IL6, THBS1*
GO:0009897	external side of plasma membrane	3.91 $\times 10^{-5}$	*THBS1*
GO:0070062	extracellular exosome	5.83 $\times 10^{-5}$	*MMP9, SPP1, THBS1*
GO:0009986	cell surface	0.001160	*THBS1*
**KEGG Pathways**			
**KEGG ID**	**KEGG function**	***p*** *-* **value**	**Associated cGBs**
hsa04151	PI3K-Akt signaling pathway	2.47 $\times 10^{-10}$	IL6, MYC, SPP1, THBS1
hsa04657	IL-17 signaling pathway	2.41 $\times 10^{-9}$	*CXCL1, MMP9, IL6*
hsa04060	Cytokine-cytokine receptor interaction	6.44 $\times 10^{-9}$	*CXCL1, IL6*
hsa04062	Chemokine signaling pathway	2.45 $\times 10^{-8}$	*CXCL1*
hsa04668	TNF signaling pathway	2.22 $\times 10^{-7}$	*MMP9, CXCL1, IL6*

#### 3.7.2 Regulatory network analysis of cGBs.

**Fig 4:**
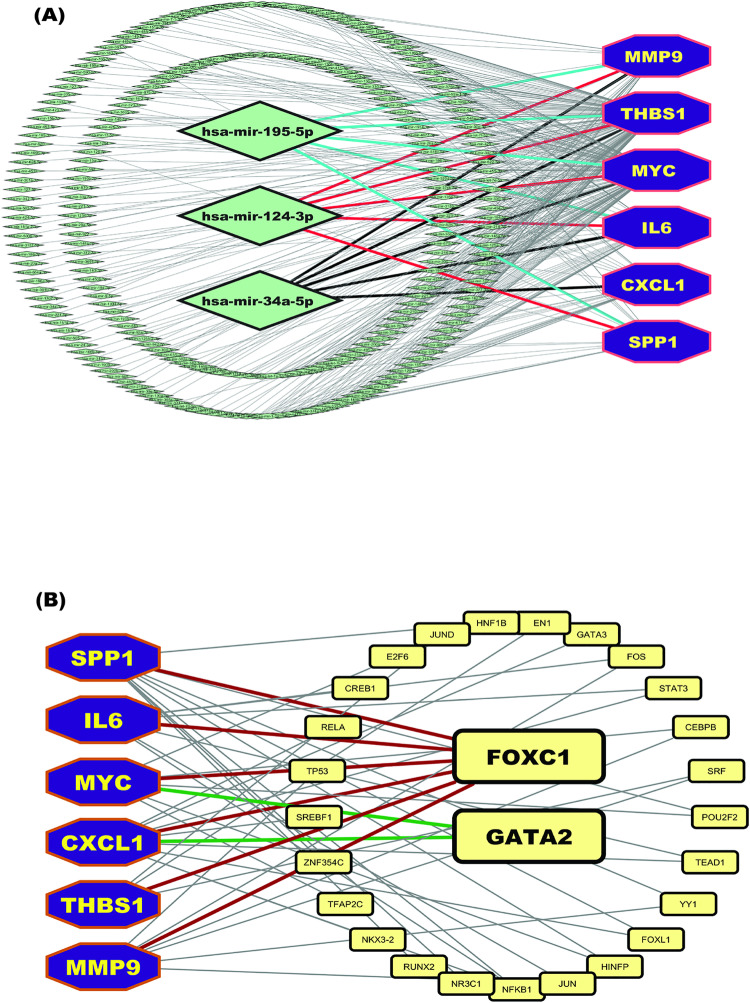
(A) cGBs -miRNA reegulatory networks. Here cGBs were marked as dark blue color with octagon shapes, miRNAs were marked as green color with diamond shape. The cGBs regulatory network with TFs proteins, where TFs were marked as yellow color with round rectangle shape and cGBs were marked as dark-blue color with octagon shape.

To identify the regulators of cGBs at both the transcriptional and post-transcriptional levels, we utilized cGBs-TF (transcription factor) and cGBs-miRNA (microRNA) regulatory networks. Our analysis focused on detecting key transcription factors and microRNAs that play significant roles in modulating the expression of gasiform the cGBs-TF regulatory network, we identified the top two transcription factors, FOXC1 and GATA2, as having the highest influence based on network centrality measures. FOXC1 and GATA2 were selected using a threshold degree of 2 and a betweenness centrality score of 34.04. These metrics indicate that FOXC1 and GATA2 are central to the regulatory network, significantly impacting the cGBs expression through transcriptional regulation. In the cGBs-miRNA regulatory network, we pinpointed three key microRNAs: hsa-mir-195-5p, hsa-mir-124-3p, and hsa-mir-34a-5p. These microRNAs were identified as the most influential based on their degree and betweenness centrality. The threshold degree for these microRNAs was set at 5, with a betweenness centrality score of 632.36. This highlights their substantial role in post-transcriptional regulation of cGBs, affecting the stability and translation of mRNA (Fig 4).

### 3.8 The cGBs-guided drug repurposing

To identify repurposable drug molecules targeting cGBs for T2D, HCC, and CRC, we performed molecular docking and ADME/T analysis. The process involved evaluating the binding interactions between drug candidates and cGB-associated proteins, followed by an assessment of the pharmacokinetic properties. The detailed methodology and findings are outlined in subsections 3.8.1 and 3.8.2.

#### 3.8.1 Exploring candidate drugs by molecular docking.

To identify repurposable drug molecules targeting cGBs, molecular docking was performed between cGB-associated receptors and selected drug candidates. The 3D structures of six receptors-MYC, MMP9, THBS1, CXCL1, IL6, and GATA2-were downloaded from the Protein Data Bank (PDB) with the codes 1a93, 6esm, 2erf, 1mgs, 1il6, and 5o9b, respectively. The FOXC1 receptor structure was obtained from the AlphaFold Protein Structure Database using UniProt ID Q12948, while the SPP1 receptor was modeled via SWISS-MODEL with UniProt ID Q9BX95.

**Fig 5:**
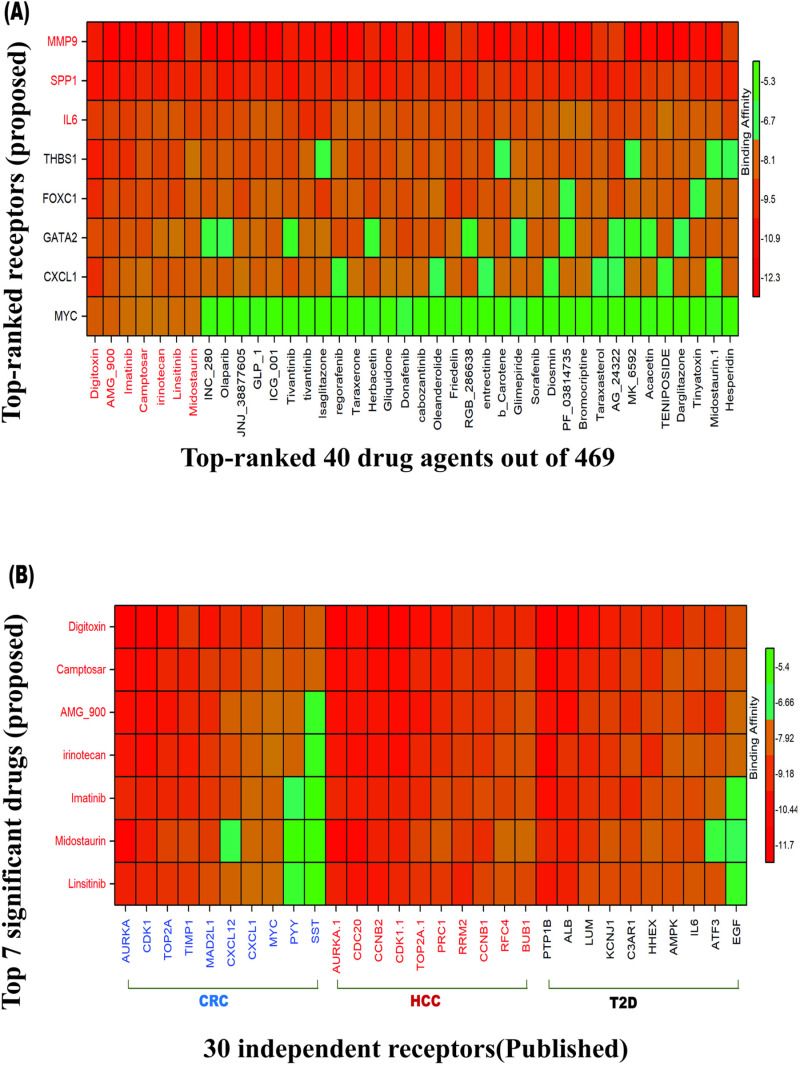
(A) The image shows the binding affinity score matrix, with the X-axis representing the top 40 drug agents (out of 469) and the Y-axis displaying the ordered proposed receptors, (B) Image of score matrix corresponding to the top 10 independent receptors for each of T2D, CRC and HCC (X-axis) and the top 7 proposed drugs (Y-axis). In X-axis, black color indicates the top 10 independent receptors of T2D, red indicates the top 10 independent receptors of HCC, blue indicates the top 10 independent receptors of CRC.

We conducted molecular docking analysis to determine the binding affinity scores (BAS) between various receptors and candidate drugs. Using these BAS values, we computed averages for both rows and columns in the BAS matrix, as detailed in S12 Table, to rank the receptors and drug candidates. Specifically, we first ranked the receptors based on the row sums of the binding affinity matrix B = (S*ij*) and then ranked the drug candidates based on the column sums. This ranking process enabled us to identify several promising drug candidates (Fig 5A).

Among these, the top seven drugs (Digitoxin, Camptosar, AMG-900, Imatinib, Irinotecan, Midostaurin, and Linsitinib) demonstrated significant binding interactions, with BAS values below -7.0 kcal/mol across all receptors. These seven drugs have emerged as potential candidates for therapeutic interventions targeting HCC, CRC and T2D simultaneously.

To verify their binding performance against other independent receptors, we considered top-ranked six cGBs for each of T2D, CRC and HCC separately, by the literature review. we collect 30 research articles separately for each of T2D, HCC and CRC, which proposed GBs that may induce T2D, HCC and CRC. Then we took into consider 10 key genomic biomarkers separately for each of CRC (S4 Table), HCC (S5 Table) and T2D (S6 Table) those were published in at least four studies. We observed that the proposed drug molecules significantly bind (BAS < -7.0 kcal/mol) with most of the independent receptors (Fig 5B & S13 Table). Therefore, the proposed cGBs-guided these top-ranked seven lead drugs could be promising as the candidate drug molecules for the treatment of CRC and HCC with T2D. To provide more information about the proposed drugs and targets, top-ranked three drug-target complexes highlighting their 3-dimension (3D) view and interacting residues were given in S14 Table.

#### 3.8.2 ADME/T analysis.

ADME/T analysis evaluates the absorption, distribution, metabolism, excretion, and potential toxicity of drug molecules, while drug-likeness assessments focus on physicochemical properties to determine their chemical suitability as therapeutic agents. The ADME/T characteristics of the selected molecules were examined to evaluate their pharmacological potential. All compounds demonstrated adequate lipophilicity, with LogP values ≤  5, and showed water solubility, as reflected by ESOL scores below -6.5. Based on Lipinski’s Rule of Five, two drugs (Imatinib and Linsitinib) complied fully with all criteria, while four others (Camptosar, Midostaurin, AMG-900, and Irinotecan) each violated one rule. One compound exhibited two violations (S15 Table). The effectiveness and safety of the proposed compounds were further assessed through ADME and toxicity evaluations. A key parameter influencing oral absorption is the Human Intestinal Absorption (HIA) score, where values above 30% indicate good absorption. Five drugs-AMG-900, Irinotecan, Camptosar, Imatinib, and Linsitinib-showed excellent HIA scores of 90% or higher, indicating high absorption in the gastrointestinal tract. The remaining two compounds, Digitoxin and Midostaurin, demonstrated favorable absorption with HIA scores of 70%. Blood-brain barrier (BBB) permeability, a crucial factor for assessing potential central nervous system (CNS) effects, was also evaluated. None of the seven compounds had a BBB permeability index above 0.3, suggesting limited BBB penetration and reduced risk of CNS toxicity. Additionally, LogPS (CNS) values indicated partial CNS availability for these compounds. The role of cytochrome P450 (CYP) enzymes, particularly CYP3A4, in drug metabolism and detoxification was examined. All proposed drugs exhibited the capacity to inhibit CYP3A4, highlighting their potential involvement in key metabolic pathways. Toxicity predictions, based on LC50 values, revealed that all compounds were non-toxic, with LC50 values exceeding 1.0 log mM. Further assessments, including AMES tests, LD50 values, and minnow toxicity evaluations, confirmed the absence of significant toxic effects.

In summary, ADME and toxicity analyses suggest that the proposed compounds have favorable pharmacokinetic and safety profiles, supporting their potential as therapeutic agents. Detailed results of these evaluations are presented in Table 4.

**Table 4:** ADME/T profile of top-ranked 7 drugs.

Compounds	Absorption			Desorption		Metabolism	Excretion	Toxicity		
	Caco2 Permeability	HIA (%)	P-gpI	BBB	CNS	CYP3A4	TC	AMES	LC50 (log mM)	LD50 (mole/kg)
				(Permeability)						
Digitoxin	0.601	74.28	Yes	-1.364	-3.21	Yes	0.44	No	3.70	3.334
Amg-900	2.975	100	Yes	-0.281	-3.156	Yes	0.13	No	2.81	2.55
Irinotecan	0.648	99.88	Yes	-1.31	-3.23	Yes	0.93	No	0.79	2.81
Imatinib	1.09	93.84	Yes	-1.37	-2.51	Yes	0.71	No	2.08	2.9
**Midostaurin**	-0.708	79.041	Yes	-0.982	-1.226	Yes	0.78	No	2.38	3.45
**Linsitinib**	1.188	93.26	Yes	-0.07	-1.86	Yes	0.73	No	-1.1	2.68
**camptosar**	0.648	99.88	Yes	-1.31	-3.23	Yes	0.93	No	0.79	2.81

According to the Drug-Likeness, and ADMET analysis of proposed compounds we conclude that, seven compounds (Digitoxin, Camptosar, AMG-900, Imatinib, irinotecan, Midostaurin and Linsitinib) would behave as a drug-like and could be used as an oral drug.

### 3.9 DFT analysis

The calculated electronic parameters for seven drug molecules provide insights into their chemical reactivity and stability. Digitoxin exhibits the largest energy gap (ΔE =  0.19659), indicating higher stability and lower reactivity compared to other drugs, while AMG_900 has the smallest gap (ΔE =  0.12683), suggesting relatively higher reactivity. Irinotecan, with the highest ionization potential (I =  0.20987), may require more energy for electron removal, indicating stable molecular orbitals. Conversely, Midostaurin shows the lowest electronegativity (χ =  0.1148), implying lesser attraction to electrons, which may influence its interaction with biological targets. The chemical hardness (η) and softness (σ) values align, showing AMG_900 as the softest compound, potentially enhancing binding flexibility. This analysis highlights the varied stability and reactivity profiles of each drug, informing their suitability in therapeutic contexts (see Table 5 and S16 Table).

**Table 5:** DFT-based electronic properties of proposed drug molecules for Reactivity and Stability Assessment Bottom of Form.

Drug	HOMO	LUMO	Energy Gap (ΔE)	Ionization Potential (I)	Electron Affinity (A)	Chemical Hardness (η)	Chemical Softness (σ)	Electron-egativity (χ)	Chemical Potential (μ)
Digitoxin	-0.206	-0.009	0.196	0.206	0.009	0.098	10.173	0.107	-0.107
AMG_900	-0.191	-0.064	0.126	0.191	0.064	0.063	15.769	0.127	-0.127
Imatinib	-0.201	-0.055	0.146	0.201	0.055	0.073	13.682	0.128	-0.128
Irinotecan	-0.209	-0.082	0.127	0.209	0.082	0.063	15.648	0.146	-0.146
Linsitinib	-0.200	-0.071	0.129	0.2001	0.071	0.064	15.500	0.135	-0.135
Midostaurin	-0.187	-0.042	0.145	0.1873	0.042	0.072	13.768	0.114	-0.114
Camptosar	-0.208	-0.072	0.136	0.2088	0.072	0.068	14.621	0.140	-0.140

### 3.10 Molecular dynamic simulation

**Fig 6:**
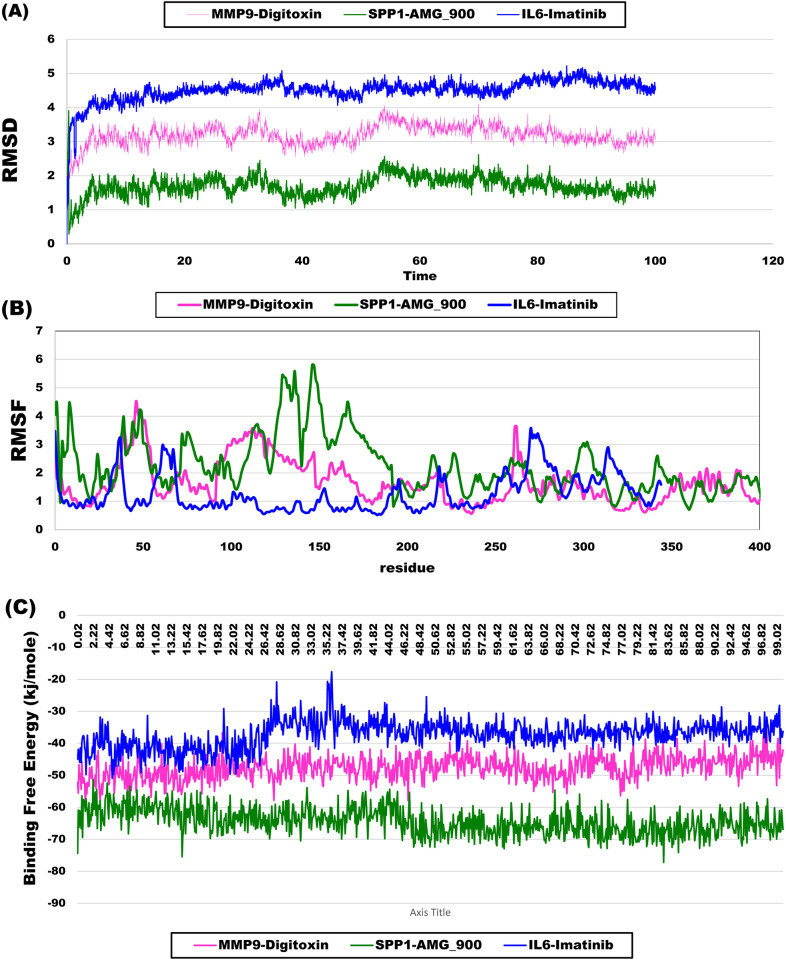
Molecular dynamics (MD) simulation results: (A) Root Mean Square Deviation (RMSD) of the backbone atoms, (B) Root Mean Square Fluctuation (RMSF) of the protein-ligand complex, and (C) Binding free energy profiles of the three complexes throughout the MD simulation.

Molecular dynamics (MD) simulations were performed to assess the stability and binding affinities of the three complexes (MMP9-DIGITOXIN, SPP1-AMG_900, and IL6-Imatinib). To evaluate the conformational change of protein structure, RMSD is considered, indicating the average distance between atoms existing at unusual sites on the target protein. The SPP1-AMG_900 complex exhibited the best stability, with an average RMSD of 1.69 Å, indicating minimal conformational changes throughout the simulation. In comparison, the MMP9-DIGITOXIN and IL6-Imatinib complexes showed average RMSD values of 3.19 Å and 4.51 Å, respectively, suggesting moderate to high fluctuations. Root mean square fluctuation (RMSF) analysis further supported these observations, with SPP1-AMG_900 displaying the highest fluctuation of 2.23 Å among the residues, while IL6-Imatinib maintained a lower fluctuation of 1.26 Å. The binding free energy calculations revealed that SPP1-AMG_900 had the most favorable interaction, with a binding free energy of -64.38 kJ/mol, compared to -47.66 kJ/mol for MMP9-DIGITOXIN and -37.23 kJ/mol for IL6-Imatinib. These results indicate that SPP1-AMG_900 is a particularly promising candidate for further investigation, showcasing enhanced stability and stronger binding affinity. Overall, the MD simulations effectively demonstrated the dynamic behavior of these complexes, supporting their potential as therapeutic options (Fig 6).

## 4. Discussion

**Fig 7:**
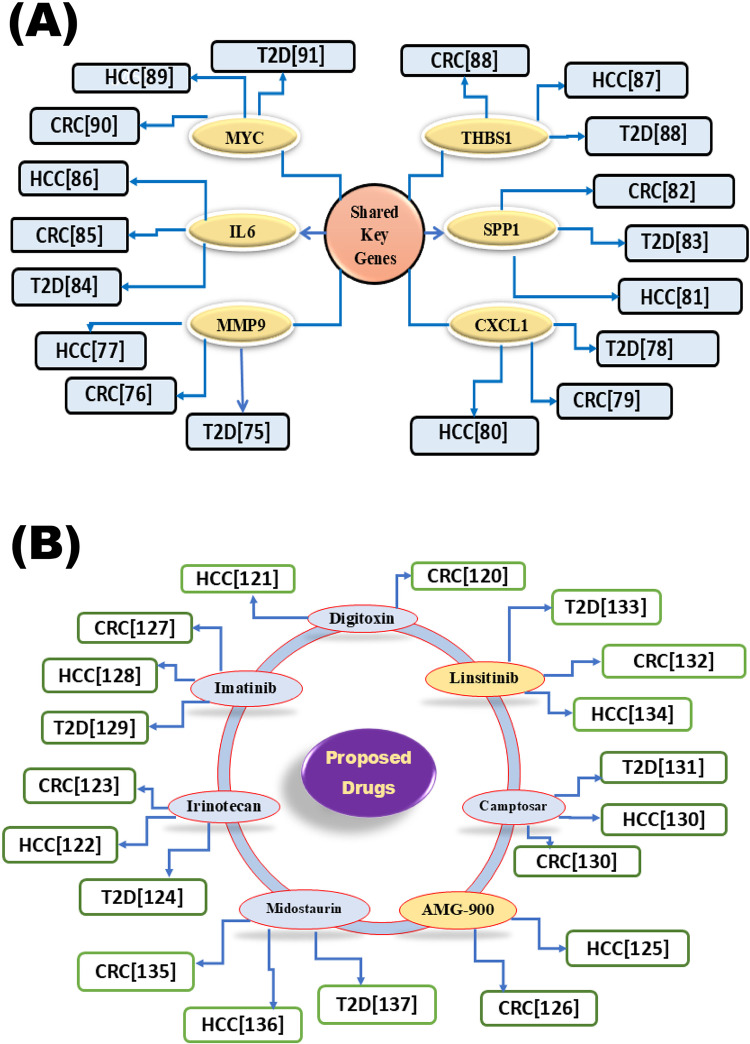
Verification of proposed cGBs and common drug molecules for T2D, HCC, and CRC through literature review. (A) Verification of cGBs and (B) Verification of drug molecules; the ellipse in green represents FDA-approved drugs; the yellow represents investigational drugs.

Population-based studies have indicated that T2D is associated with CRC [73] and HCC [74]. Therefore, in this study, we investigated their genetic association based on common differentially expressed genes (cDEGs) and detected top-ranked 6 cDEGs (*MYC, MMP9, IL6, THBS1, CXCL1*, and *SPP1*) as those three disease-causing cGBs for exploring their common drug molecules (Fig 3). The association of cGBs with T2D, HCC, and CRC was verified through the literature review, disease-cGB interaction analysis and expression analysis of cGBs with T2D, HCC, and CRC, functional enrichment analysis with GO-terms and KEGG-pathways, and regulatory network analysis with transcription factors (TFs) as discussed below. The association of these six cGBs with T2D, CRC, and HCC is further supported by previous individual studies, including those involving *MYC* [75–77], *CXCL1* [78–80], *SPP1* [81–83], *IL6* [84–86], *THBS1* [87–89], and *MMP9* [90–92] as displayed in Fig 7A. We conducted a regulatory network analysis to identify key transcription factors (TFs) associated with cGBs that are crucial for disease development. In this analysis, we highlighted two main TFs (GATA2 and FOXC1) that are significantly connected with the cGBs. Subsequently, we performed an enrichment analysis on the cGBs to uncover critical biological processes (BP), molecular functions (MF), and KEGG pathways associated with the development of T2D, CRC, and HCC. The results, as shown in Table 3, indicated that these diseases are notably enriched in various essential biological processes and pathways.

*MYC* is a critical oncogene within the positive regulation of cell proliferation and the PI3K-Akt signaling pathway, driving unchecked cell division and metabolic reprogramming in CRC and HCC [93]. In T2D, its dysregulation disrupts normal glucose metabolism, impacting insulin sensitivity and pancreatic β-cell function. By promoting glycolysis and other metabolic shifts, it creates a cellular environment that not only supports rapid cancer cell proliferation but also disrupts glucose homeostasis in T2D [94]. Its influence extends to signal transduction pathways, where it drives both the growth of cancerous cells and the metabolic adaptations characteristic of diabetes [95]. MMP9’s role in the extracellular matrix (ECM) degradation is vital for the pathological progression of T2D, CRC, and HCC [96]. In T2D, elevated levels degrade ECM components in blood vessels, contributing to vascular complications and atherosclerosis [97]. In cancers like CRC and HCC, it facilitates metastatic spread by breaking down ECM barriers, which allows cancer cells to invade adjacent tissues [98]. This gene’s activity in the TNF signaling pathway further supports inflammation, which is common to both metabolic and cancerous diseases, emphasizing its role in fostering an environment that enables cancer cell migration and metastasis [99]. *IL6* is a central cytokine in the *cytokine-cytokine receptor interaction* and *IL-17 signaling pathways,* where it orchestrates immune responses and maintains chronic inflammation*,* a key factor in both T2D and cancer*.* In T2D, it persistently activates the JAK/STAT pathway, promoting insulin resistance and metabolic disruption [100]. This role extends to CRC and HCC, where it supports tumor growth by enhancing cell survival, proliferation, and metastasis through the PI3K-Akt *signaling pathway*, which inhibits apoptosis and drives tumor progression [101]. Functioning in the *inflammatory response* and *cytokine activity*, *IL6* acts systemically from the *extracellular space,* regulating inflammation that contributes to insulin resistance in T2D and creates a pro-tumorigenic environment in cancers [102]. By bridging immune, metabolic, and oncogenic processes, it exemplifies how chronic inflammation can serve as a foundation for both metabolic and cancerous pathologies. CXCL1 is involved in immune cell recruitment via the *chemokine-mediated signaling pathway*. The *chemokine-mediated signaling pathway* facilitates the recruitment of immune cells, such as macrophages and T cells, to tissues under stress or injury. This mechanism fosters a pro-inflammatory environment in adipose tissue, liver, and skeletal muscles, contributing to systemic insulin resistance in T2D [103]. In CRC and HCC, CXCL1 fosters a tumor-promoting inflammatory environment by supporting cancer cell survival and proliferation [104]. Through the chemokine and cytokine receptor interaction pathways, CXCL1 establishes crosstalk between cancer cells and inflammatory cells, ultimately supporting cellular environments where both insulin resistance and cancer cell invasiveness can thrive [105]. Thrombospondin-1 (THBS1) is an extracellular matrix glycoprotein that modulates immune responses, cell adhesion, and angiogenesis, significantly impacting T2D and cancer. THBS1 promotes vascular inflammation by activating inflammatory pathways in the endothelium, leading to endothelial dysfunction. This disruption impairs insulin signaling, contributing to the development of insulin resistance in T2D. [106]. The gene (THBS1) also facilitates cancer cell survival and tumor progression by creating a pro-angiogenic and immunosuppressive microenvironment. The pro-angiogenic activity of THBS1 promotes the formation of new blood vessels, ensuring an adequate supply of oxygen and nutrients to the growing tumor [107]. It is active in the *PI3K-Akt signaling pathway*, reinforcing survival signals within the tumor microenvironment, which is essential for tumor sustenance and immune evasion [108]. SPP1, or osteopontin, is a multifunctional protein linked to the *NF-kappa B signaling pathway*, which regulates inflammation and cell survival [109]. Its inflammatory role disrupts insulin sensitivity, contributing to the progression of T2D [110]. Additionally, it supports immune evasion and tumor growth by creating a tumor-promoting microenvironment in CRC and HCC. Located in the extracellular matrix, SPP1 is essential for tissue remodeling and immune cell modulation. By bridging chronic inflammation and immune regulation, SPP1 connects metabolic disorders such as T2D to cancer progression, facilitating tumor survival and metastasis [111]. The cellular response to lipopolysaccharide (LPS), a bacterial endotoxin, further exacerbates this condition. LPS activates receptors on immune and non-immune cells, triggering signaling cascades that lead to the release of pro-inflammatory cytokines and chemokines [112,113]. This response amplifies inflammation, disrupting insulin signaling pathways and reducing the sensitivity of insulin receptors[114]. The inflammatory response acts as the overarching mechanism, with chronic low-grade inflammation causing beta-cell dysfunction, impairing insulin secretion, and increasing oxidative stress. This persistent inflammatory state also promotes glucose intolerance and metabolic imbalances, creating a vicious cycle that worsens T2D progression [115]. Collectively, these processes highlight the complex interplay between immune dysregulation, inflammation, and metabolic dysfunction in T2D pathology.

FOXC1 and GATA2 regulate all cGBs (*MYC, MMP9, THBS1, IL6, CXCL1, SPP1*) associated with T2D, CRC, and HCC. FOXC1 contributes to insulin resistance by driving chronic inflammation and promotes cancer progression by enhancing cell proliferation, survival, invasion, and a pro-tumorigenic microenvironment [116,117]. Similarly, GATA2 plays a multifaceted role by exacerbating metabolic dysfunction through inflammatory responses, while also facilitating cancer cell invasion, survival, angiogenesis, and tumor growth. Its modulation of cGBs further supports tissue remodeling and the tumor microenvironment across these diseases [118,119]. These findings highlight the intricate network of interactions and pathways contributing to the pathogenesis of T2D, CRC, and HCC.

The Random Forest (RF)-based prediction model effectively differentiated between the three diseases and control groups based on cGB expression. With a high predictive accuracy (AUC >  0.92 and ACC >  0.81), the model highlights the importance of cGBs in disease classification (S7 Fig, S11 Table). The box-plots analysis showed that 6 cGBs are significantly upregulated in T2D, CRC, and HCC compared to the control groups, which supported our results (S6 Fig).

To explore cGB-guided possible pharmacological treatments for HCC, CRC, and T2D, we estimated the binding affinity scores between 469 meta-drug molecules and cGB-mediated receptors. We then identified the top seven drug agents (Digitoxin, Camptosar, AMG-900, Imatinib, Irinotecan, Midostaurin, and Linsitinib) as the most promising repurposable candidate drugs for HCC, CRC, and T2D. We performed molecular docking analysis (Fig 5, S12 Table & S13 Table) to find possible drug candidates for the treatment of HCC, CRC, and T2D. Digitoxin, Camptosar, AMG-900, Imatinib, Irinotecan, Midostaurin, and Linsitinib were the seven candidate drugs out of the 469 that were ranked top that showed significant binding affinities with the target proteins (cGBs and their TFs). Subsequently, we compared the effectiveness of these seven chemical molecules against 10 independent receptors separately for each of T2D, HCC, and CRC (published by others) that supported our results. We also used drug-likeness and ADME/T analysis to assess the suggested drug molecules effectiveness. Each of the identified drug molecules satisfied at least four rules of Lipinski’s rule of five, demonstrating their drug-like properties (S14 Table). The seven drug molecules that were chosen for analysis showed favorable ADME/T profiles, high HIA percentages ranging from 68% to 99.88%, sufficient water solubility, and no carcinogenic characteristics. Molecular dynamics simulations of the top three drug candidates demonstrated stable interactions with their target proteins. These findings underscore the stability of the drug-protein complexes and reinforce their potential as therapeutic agents, indicating that these drugs hold promise as effective treatment options. The literature review provided further support for the potential effectiveness of our suggested drugs as treatments for T2D, CRC, and HCC individually. Digitoxin [120,121], Irinotecan [122–124], AMG-900 [125,126], Imatinib [127–129], Camptosar [130,131], Linsitinib [132–134], and Midostaurin [135–137], as displayed in (Fig 7B). All of our proposed drugs were validated as common candidate molecules for T2D, CRC, and HCC by individual studies focused on these diseases. According to the DrugBank (DB) database, Digitoxin (ID-DB01396), Irinotecan (ID-DB00762), Imatinib (ID-DB00619), and Midostaurin (ID-DB06579) are FDA-approved for the treatment of various cancers and other diseases. Digitoxin, a cardiac glycoside, is widely used in treating congestive heart failure, arrhythmias, and cardiac insufficiency [138]. Beyond its cardiovascular applications, Digitoxin has shown promise as an effective anticancer agent, particularly in treating cetuximab-resistant CRC and HCC [139]. Imatinib, a well-known FDA-approved anticancer drug cataloged in DrugBank, is primarily prescribed for chronic myeloid leukemia (CML) and gastrointestinal stromal tumors (GIST) [140]. Interestingly, Imatinib has also demonstrated potential in improving glycemic control in diabetic patients, suggesting it may influence glucose-regulating metabolic pathways beyond its conventional cancer applications [129]. Studies have further highlighted Imatinib’s ability to inhibit tumor growth in xenograft models derived from HCC patients, indicating its broader application in targeting aggressive tumor types [128]. Collectively, these findings underscore Imatinib’s versatile therapeutic impact across multiple pathological conditions. Irinotecan, a chemotherapy agent primarily used to treat colorectal cancer, is being investigated for its potential in managing HCC, particularly in patients with cirrhosis. When administered via intra-arterial infusion at a recommended dose of 25 mg/m2/day, Irinotecan has shown feasibility for HCC treatment [141]. Combining Irinotecan with metformin, a common T2D medication, may offer a promising approach for CRC patients who also have T2D [142]. Linsitinib, a small molecule that inhibits both insulin receptor tyrosine kinase and IGF-1 receptor activities, has shown preclinical efficacy as a standalone or combination treatment for CRC [143]. It has also been explored in Phase II clinical trials for advanced HCC, further indicating its therapeutic promise [134]. Midostaurin’s potent inhibition of protein kinase C and vascular endothelial growth factor receptor makes it a valuable option for diabetic retinopathy, given its role in mitigating key angiogenic factors [137]. Notably, studies have also demonstrated that midostaurin can inhibit CRC cell growth, causing structural changes such as multinucleation and micronuclei formation, highlighting its anticancer potential [135]. Therefore, the findings of this study could provide valuable resources for the diagnosis and treatment of T2D, HCC, and CRC simultaneously. However, a limitation of the study is that the identified cGBs and candidate therapeutic agents for these diseases have not yet been experimentally validated.

## 5. Conclusion

This study identified six common genomic biomarkers (cGBs) - *MYC, MMP9, IL6, THBS1, SPP1*, and *CXCL1*-that differentiate patients with T2D, HCC, and CRC from control groups. The differential expression patterns of these genes were validated using independent datasets from the NCBI, TCGA, and GTEx databases. Through cGBs set enrichment analysis, we identified significant common biological processes, molecular functions, and pathways linked to the development of T2D, HCC, and CRC. Additionally, regulatory network analysis revealed several transcription factors as the regulators of these cGBs. Further, seven top-ranked candidate drug agents (Digitoxin, Camptosar, AMG-900, Imatinib, Irinotecan, Midostaurin, and Linsitinib) were proposed as potential treatments, guided by molecular docking, drug-likeness assessments, and ADME/T analysis. Literature review also supported the validity of these drug targets and agents, confirming their relevance as common receptor proteins and agents for each of T2D, HCC, and CRC. However, experimental validation is necessary to confirm these results and their clinical applicability.

Therefore, our findings provide a promising foundation for developing an effective treatment strategy for patients with HCC and CRC who also have T2D.

## Supporting information

S1 Fig Sample clustering to detect outlier. (A) GSE9348; (B) GSE121248; (C) GSE76896. All samples are located in the clusters and pass the cutoff thresholds. (DOCX)

S2 Fig Analysis of network topology for selecting soft-thresholding powers. The left panel depicts the Scale-free fit index for different powers (β). The right panel depicts the Mean connectivity analysis for various soft-thresholding powers (β). The power when the correlation is required to reach 0.84 is used as the β value. In case of (A) GSE9348 it was 11; (B) GSE121248 it was 15, and (C) GSE76896 it was 14. (DOCX)

S3 Fig Clustering of module eigengene for merging close modules. Cut height of module eigengene was set to 0.17 for each dataset. (DOCX)

S4 Fig The correlation values and associated P-values (in parenthesis) were used to indicate the module-trait relations, and a wide range of colors were used to represent them. Module Eigengenes (MEs) are displayed in the rows, and the column indicates trait (CRC/HCC/T2D). Blue, turquoise, grey modules from (A) GSE9348; turquoise, grey, yellow from (B) GSE121248; and Salmon, Pink, Brown, Blue and Green modules from (C) GSE76896; had significant correlation with trait. (DOCX)

S5 Fig Heatmap for upregulated and downregulated cDEGs. It illustrates the expression levels of upregulated and downregulated common DEGs (cDEGs). Red indicates low gene expression, while green represents high gene expression across the samples. (DOCX)

S6 Fig Expression patterns of cGBs with Boxplots. (A) Expression patterns of cGBs with Boxplots for Colorectal cancer and Hepatocellular carcinoma (B) Expression patterns of cGBs with Boxplots for type 2 diabetes. (DOCX)

S7 Fig ROC curve illustrating the effectiveness of the RF-based prediction model with cGBs. Blue indicates the performance with the training dataset, and red indicates the performance with the test datasets in A, B and C. (DOCX)

S1 Table Collection of candidate drug agents for CRC from published articles and additional sources. (DOCX)

S2 Table Collection of candidate drug agents for HCC from published articles and additional sources. (DOCX)

S3 Table Collection of candidate drug agents for T2D from published articles and additional sources. (DOCX)

S4 Table Collection of Colorectal cancer (CRC) causing KGs from different published articles to select top-ranked publicly available receptors. (DOCX)

S5 Table Collection of Hepatocellular causing (HCC) causing KGs from different published articles to select top-ranked publicly available receptors. (DOCX)

S6 Table Collection of Type 2 diabetes (T2D) causing KGs from different published articles to select top-ranked publicly available receptors. (DOCX)

S7 Table List of cDEGs, including upregulated and downregulated genes, among CRC, HCC, and T2D. (DOCX)

S8 Table aLog2FC values of cDEGS in T2D, HCC and CRC. (DOCX)

S9 Table List of cGBs from PPI network based on different topological measures. (DOCX)

S10 Table Associations of cGBs with various diseases. (DOCX)

S11 Table Performance scores of the Random Forest-based prediction model. (DOCX)

S12 Table Docking (binding affinity) scores (kcal/mol) between the proposed target genes/proteins (receptors) and the top-ranked candidate drugs. (DOCX)

S13 Table Docking (binding affinity) scores (kcal/mol) between the published target genes/proteins (receptors) and the top seven proposed drugs. (DOCX)

S14 Table 3D visualization of strong binding interactions between target proteins and drugs. (DOCX)

S15 Table Drug-likeness profiles of the top ranked 7 drugs. (DOCX)

S16 Table Frontier molecular orbitals diagram for HOMO and LUMO of proposed drug compounds. (DOCX)
