# Supplementary material for: Exploring common genomic biomarkers to disclose common drugs for the treatment of colorectal cancer and hepatocellular carcinoma with type-2 diabetes through transcriptomics analysis
Source: PLoS One. 2025 Mar 24;20(3):e0319028. doi: 10.1371/journal.pone.0319028 (PMC11932495; doi:10.1371/journal.pone.0319028)
Supplement: S1 Fig — (DOCX) [file pone.0319028.s001.docx]

**
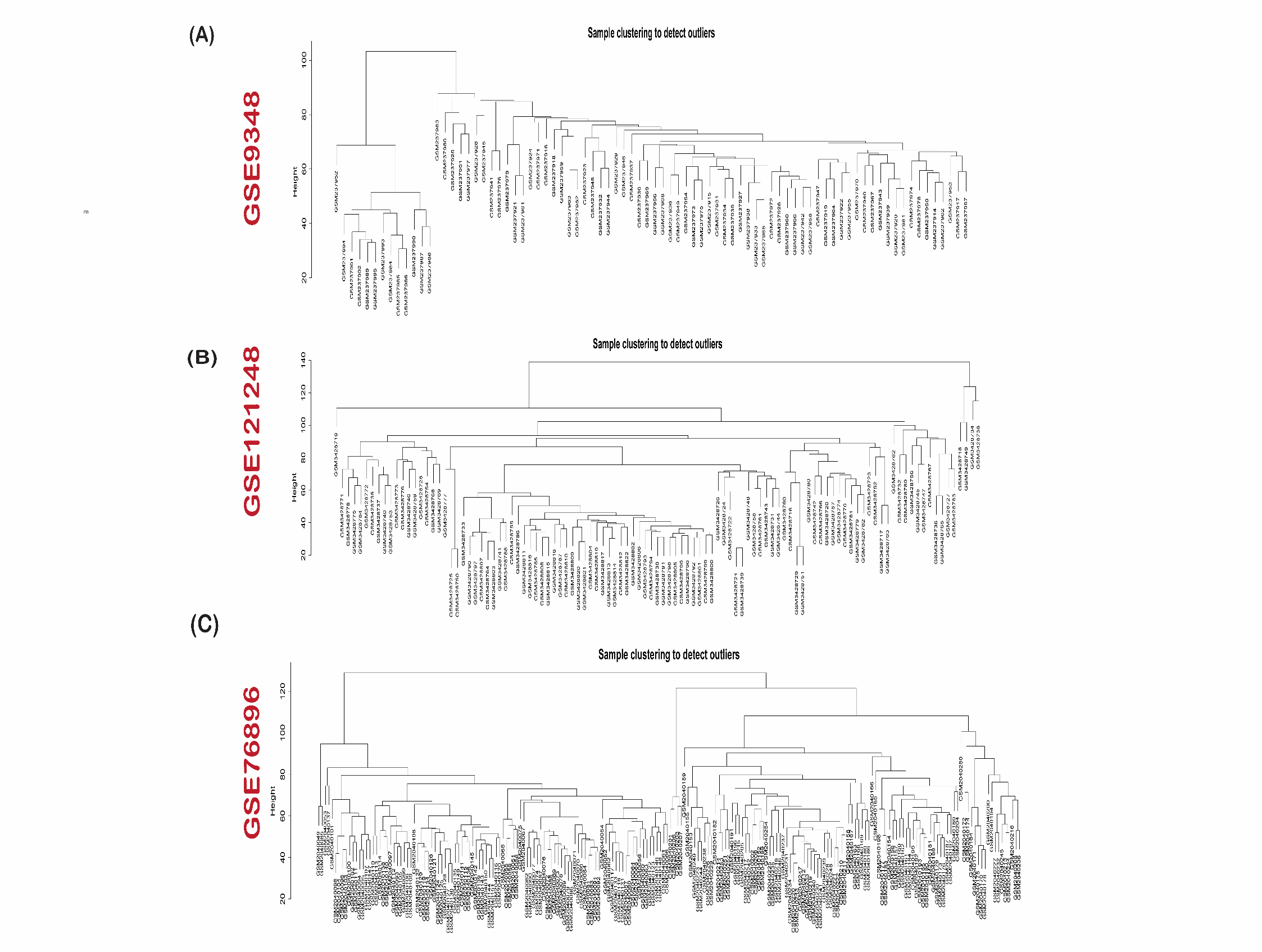
**

**S1 Fig. Sample clustering to detect outlier. (A) GSE9348; (B) GSE121248; (C) GSE76896. All samples are located in the clusters and pass the cutoff thresholds**
