# Supplementary material for: Exploring common genomic biomarkers to disclose common drugs for the treatment of colorectal cancer and hepatocellular carcinoma with type-2 diabetes through transcriptomics analysis
Source: PLoS One. 2025 Mar 24;20(3):e0319028. doi: 10.1371/journal.pone.0319028 (PMC11932495; doi:10.1371/journal.pone.0319028)
Supplement: S2 Fig — (DOCX) [file pone.0319028.s002.docx]

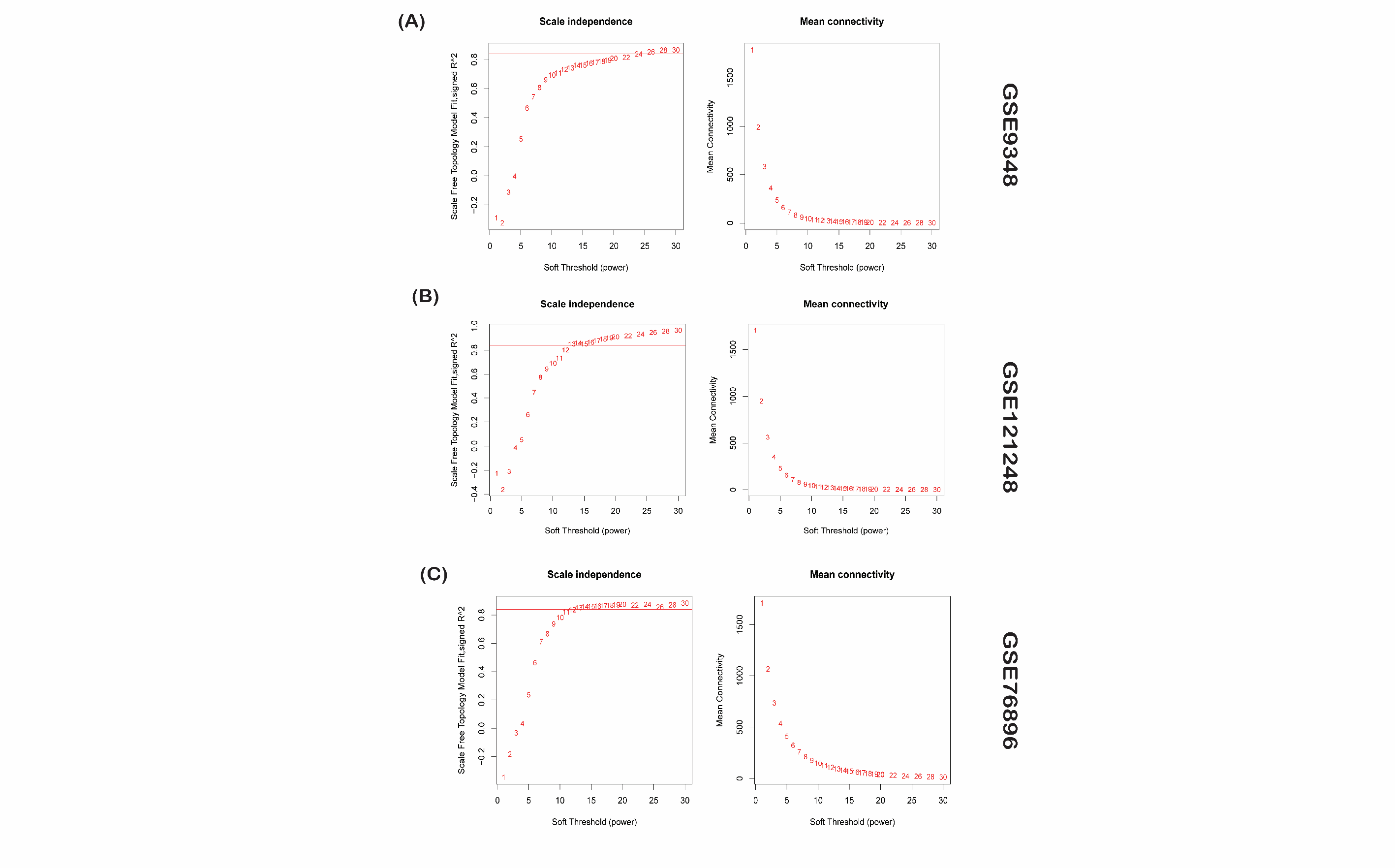


**S2 Fig. Analysis of network topology for selecting soft-thresholding powers. The left panel depicts the Scale-free fit index for different powers (β). The right panel depicts the Mean connectivity analysis for various soft-thresholding powers (β). The power when the correlation is required to reach 0.84 is used as the β value. In case of (A) GSE9348 it was 11; (B) GSE121248 it was 15, and (C) GSE76896 it was 14.**
