# Supplementary material for: Exploring common genomic biomarkers to disclose common drugs for the treatment of colorectal cancer and hepatocellular carcinoma with type-2 diabetes through transcriptomics analysis
Source: PLoS One. 2025 Mar 24;20(3):e0319028. doi: 10.1371/journal.pone.0319028 (PMC11932495; doi:10.1371/journal.pone.0319028)
Supplement: S3 Fig — (DOCX) [file pone.0319028.s003.docx]

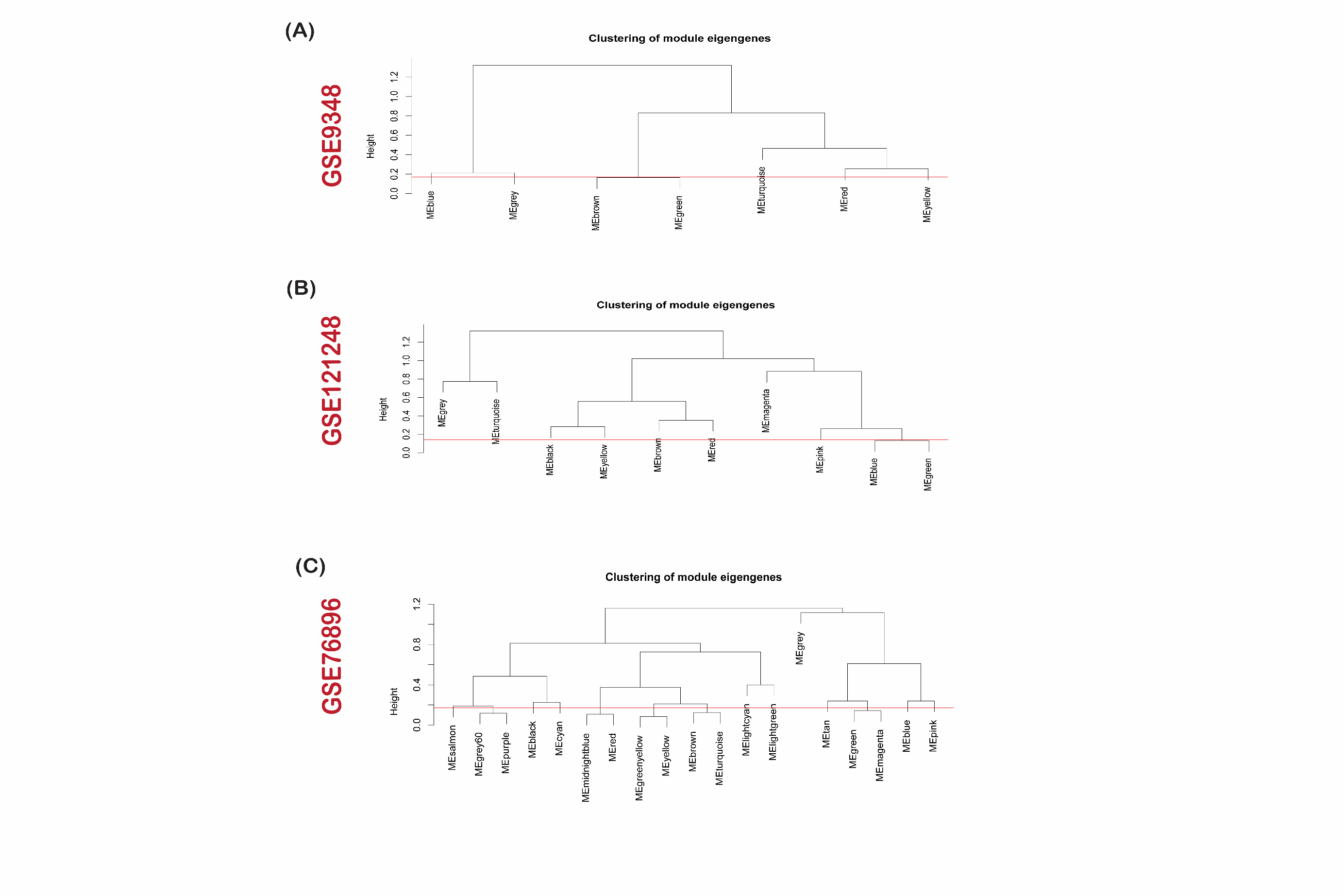


**S3 Fig. Clustering of module eigengene for merging close modules. Cut height of module eigengene was set to 0.17 for each dataset.**
