# Supplementary material for: Exploring common genomic biomarkers to disclose common drugs for the treatment of colorectal cancer and hepatocellular carcinoma with type-2 diabetes through transcriptomics analysis
Source: PLoS One. 2025 Mar 24;20(3):e0319028. doi: 10.1371/journal.pone.0319028 (PMC11932495; doi:10.1371/journal.pone.0319028)
Supplement: S4 Fig — (DOCX) [file pone.0319028.s004.docx]

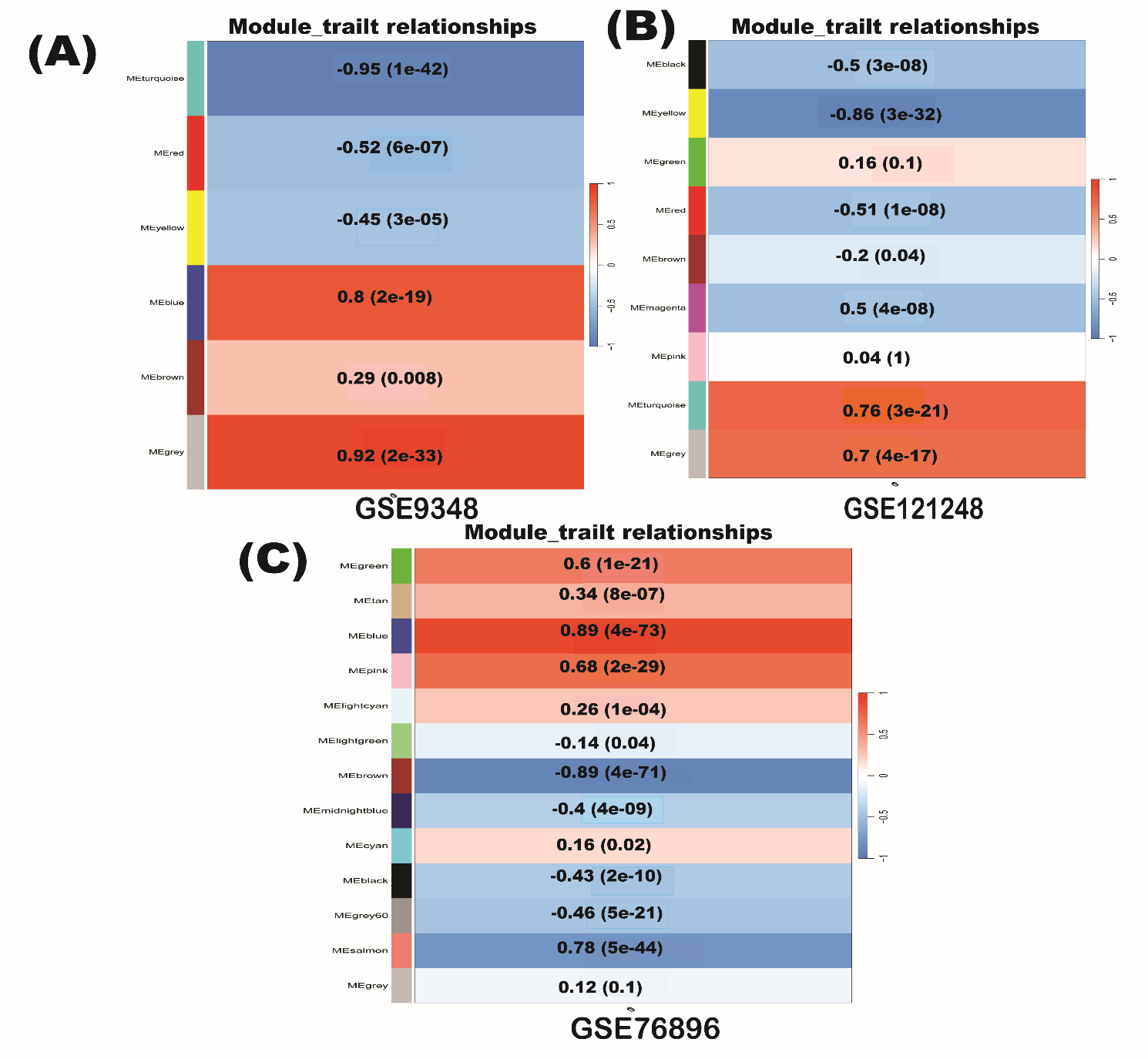


**S4 Fig. The correlation values and associated P-values (in parenthesis) were used to indicate the module-trait relations, and a wide range of colors were used to represent them. Module Eigengenes (MEs) are displayed in the rows, and the column indicates trait (CRC /HCC/T2D). Blue, turquoise, grey modules from (A) GSE9348; turquoise, grey, yellow from (B) GSE121248; and Salmon, Pink, Brown, Blue and Green modules from (C) GSE76896; had significant correlation with trait.**
