# Supplementary material for: Exploring common genomic biomarkers to disclose common drugs for the treatment of colorectal cancer and hepatocellular carcinoma with type-2 diabetes through transcriptomics analysis
Source: PLoS One. 2025 Mar 24;20(3):e0319028. doi: 10.1371/journal.pone.0319028 (PMC11932495; doi:10.1371/journal.pone.0319028)
Supplement: S5 Fig — (DOCX) [file pone.0319028.s005.docx]

**
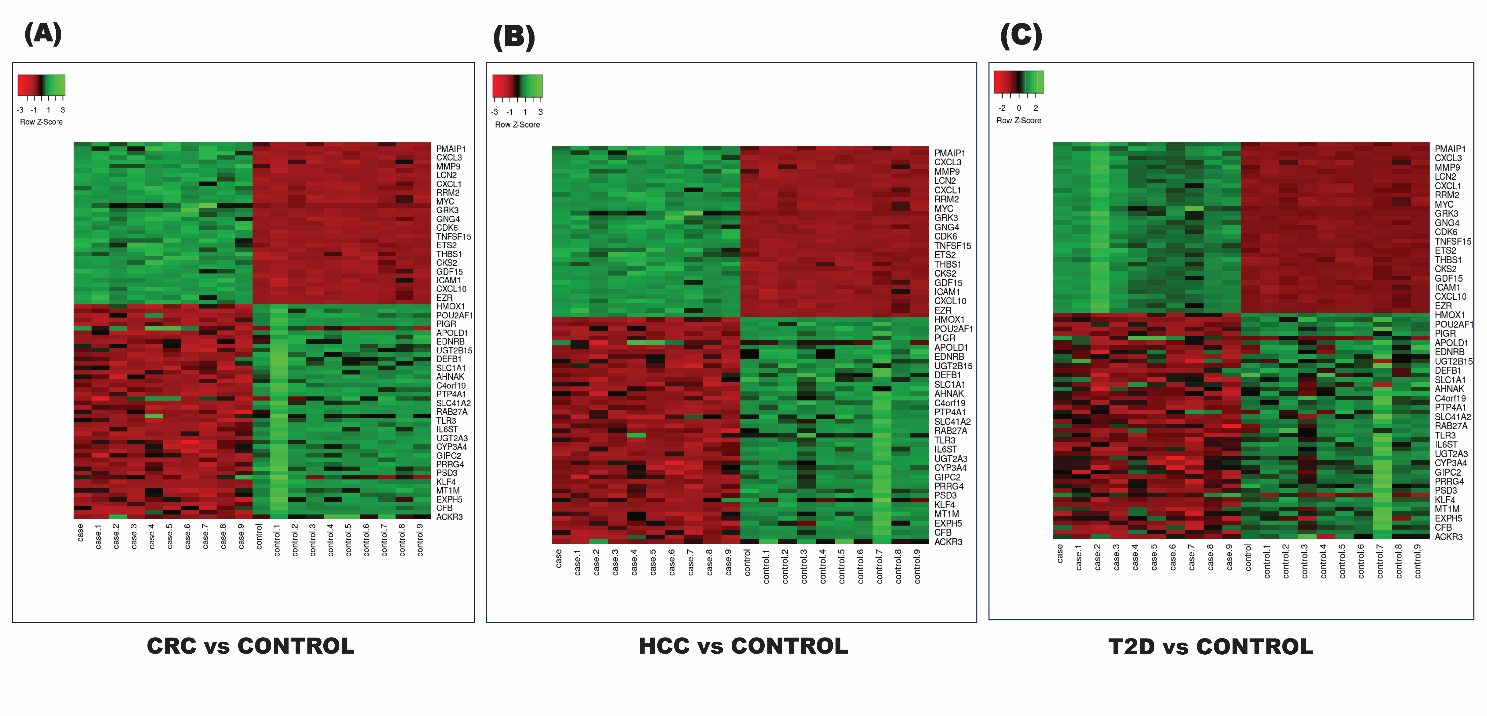
**

**S5 Fig. Heatmap for upregulated and downregulated cDEGs. It illustrates the expression levels of upregulated and downregulated common DEGs (cDEGs). Red indicates low gene expression, while green represents high gene expression across the samples.**
