# Supplementary material for: Exploring common genomic biomarkers to disclose common drugs for the treatment of colorectal cancer and hepatocellular carcinoma with type-2 diabetes through transcriptomics analysis
Source: PLoS One. 2025 Mar 24;20(3):e0319028. doi: 10.1371/journal.pone.0319028 (PMC11932495; doi:10.1371/journal.pone.0319028)
Supplement: S6 Fig — (DOCX) [file pone.0319028.s006.docx]

(A)


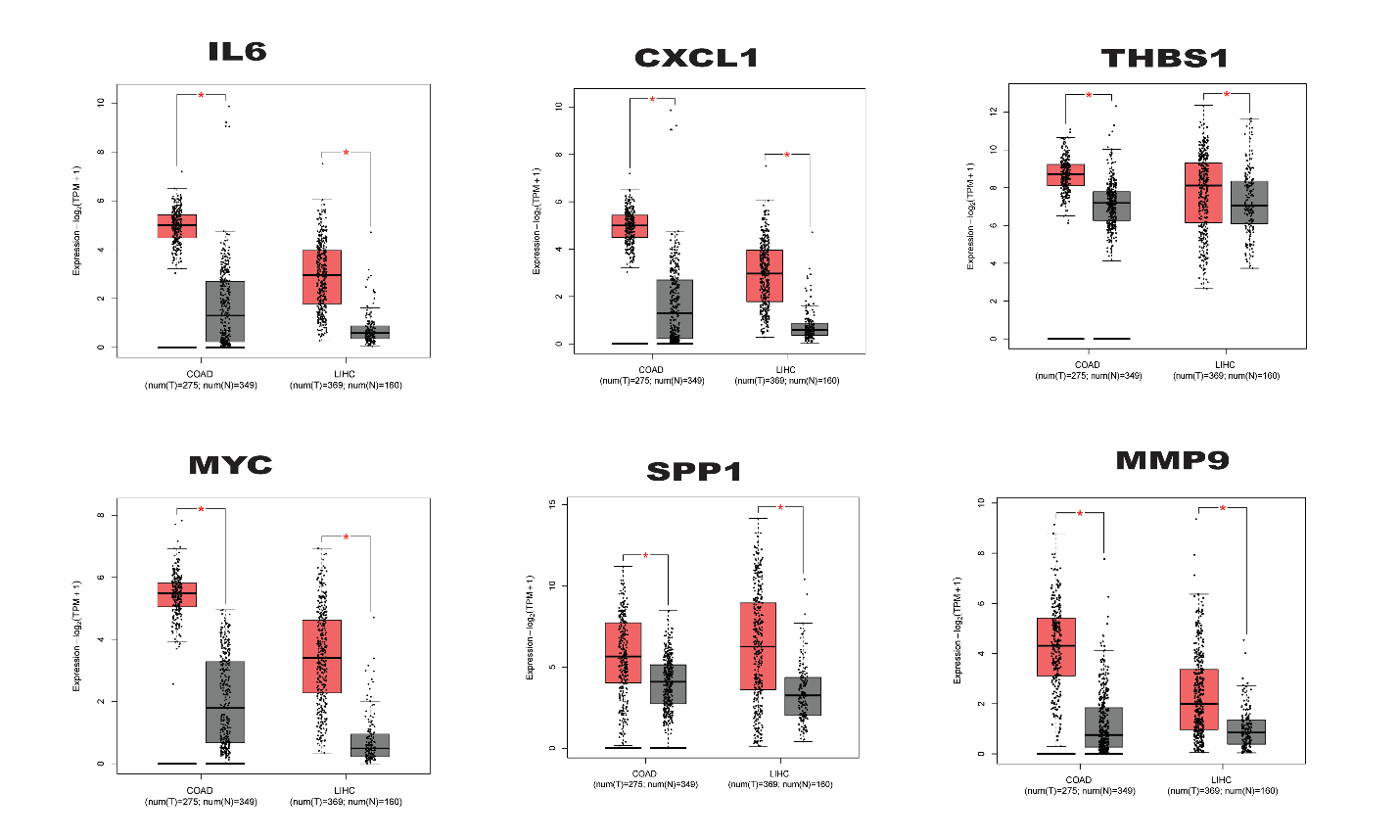


(B)


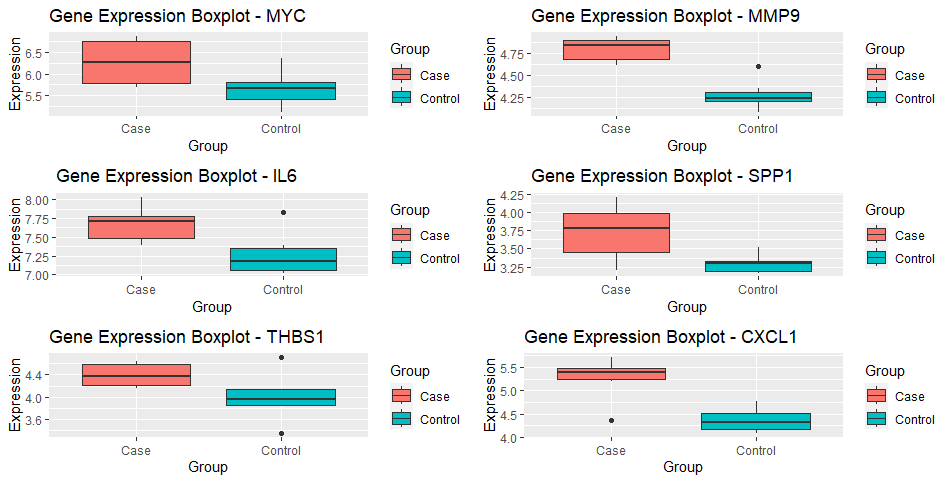


**S6 Fig. Expression patterns of cGBs with Boxplots.** **(A) Expression patterns of cGBs with Boxplots for Colorectal cancer and Hepatocellular carcinoma (B) Expression patterns of cGBs with Boxplots for type 2 diabetes**
