# Supplementary material for: Exploring common genomic biomarkers to disclose common drugs for the treatment of colorectal cancer and hepatocellular carcinoma with type-2 diabetes through transcriptomics analysis
Source: PLoS One. 2025 Mar 24;20(3):e0319028. doi: 10.1371/journal.pone.0319028 (PMC11932495; doi:10.1371/journal.pone.0319028)
Supplement: S7 Fig — (DOCX) [file pone.0319028.s007.docx]

**
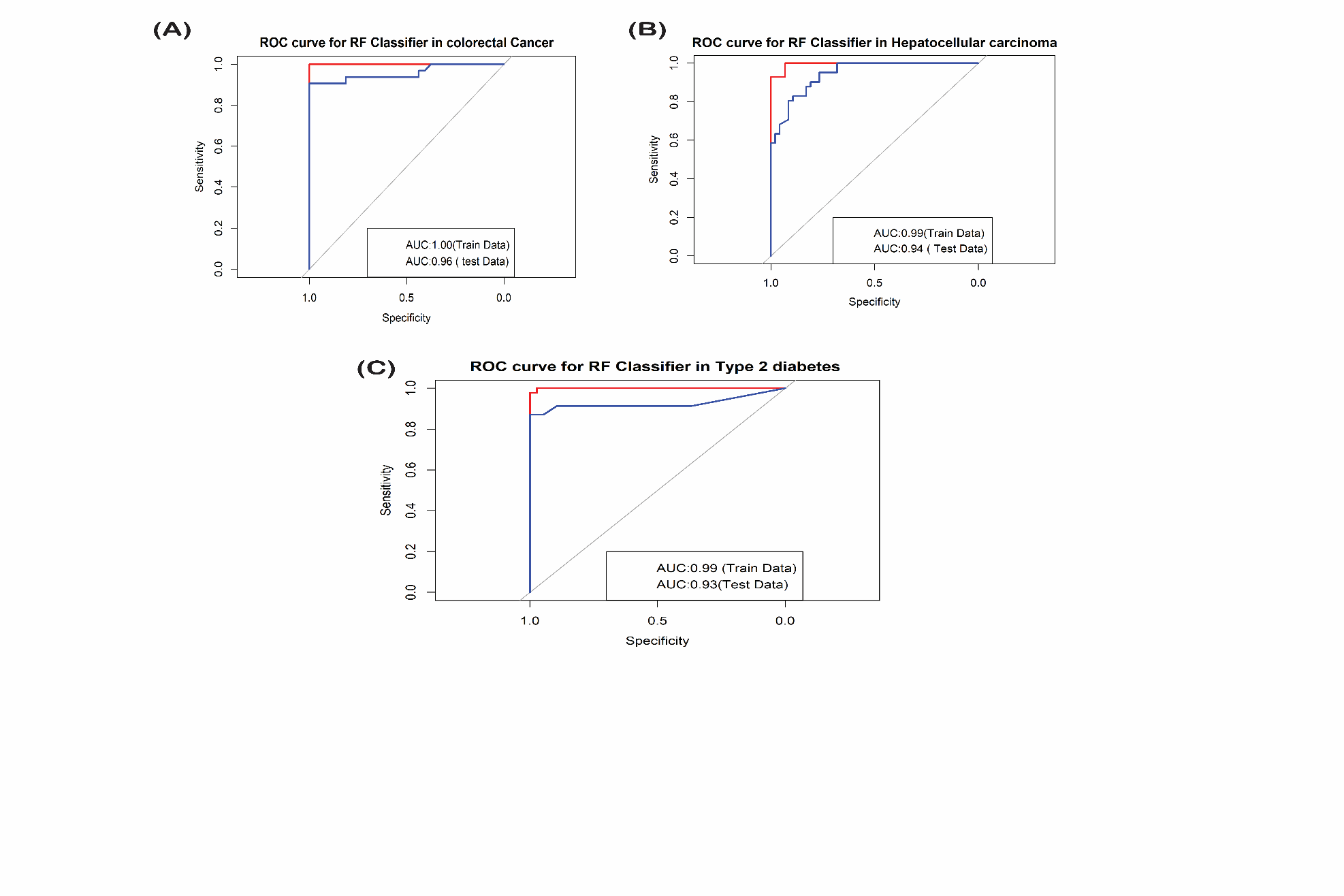
**

**S7 Fig. ROC curve illustrating the effectiveness of the RF-based prediction model with cGBs. Blue indicates the performance with the training dataset, and red indicates the performance with the test datasets in A, B and C**
