# Supplementary material for: Exploring common genomic biomarkers to disclose common drugs for the treatment of colorectal cancer and hepatocellular carcinoma with type-2 diabetes through transcriptomics analysis
Source: PLoS One. 2025 Mar 24;20(3):e0319028. doi: 10.1371/journal.pone.0319028 (PMC11932495; doi:10.1371/journal.pone.0319028)
Supplement: S1 Table — (DOCX) [file pone.0319028.s008.docx]

| **S1 Table: Collection of candidate drug agents for CRC from published articles and additional sources.** | |
| --- | --- |
| Articles | Drug agents |
| Loupakis et al., 2014[11] | Irinotecan,oxaliplatin |
| Vodenkova et al., 2020[12] | oral 5-FU |
| Maroun, 2001[13] | capecitabine |
| Tirendi et al., 2023[14] | (S-1), trifluridine/tipiracil (FTD/TPI, formerly TAS-102), regorafenib, Eniluracil (5-ethynyluracil), Tegafur |
| Ross et al., 2010[15] | gemcitabine |
| Teixeira-Guedes et al., 2022[16] | Cisplatin, carboplatin |
| C., 2017[17] | Masitinib, Pembrolizumab, Atezolizumab |
| Geng et al., 2017[18] | Cetuximab, panitumumab, Aflibercept, Regorafenib, Ramucirumab, Apatinib, Fruquintinib, Vemurafenib, Trametinib, Selumetinib, Tivantinib, Rilotumumab, Pembrolizumab, INC280, Famitinib,  Sym004, LGX818, Novartis, Nivolumab |
| Arbuck, 1989[19] | Leucovorin |
| Ferrández et al., 2012[20] | aspirin |
| American Cancer Society, 2018[21] | Ziv-aflibercept, Encorafenib, Trastuzumab,Tucatinib, Lapatinib, Fam-trastuzumab deruxtecan, Larotrectinib, entrectinib, Regorafenib |
| Cancer.Net, 2021[22] | Dostarlimab, ipilimumab |
| drugs.com | Keytruda, Zaltrap, Lonsurf, Eloxatin, Camptosar, Vectibix, Erbitux, Stivarga, fluorouracil, Abysms, Avzivi, Fruzaqla, Vegzelmainterferon beta-1b, Khapzory, levoleucovorin, trifluridine, Zirabev, Braftovi,Cyramza, Yervoy, Mvasi,Betaseron,Opdivo, Fusilev |
| Wang & DuBois, 2013[23] | NSAIDs, celecoxib, COXIBs |
| DGIDB | Methylprednisolone, Quinapril, Prednisone,Glutamine , Amifostine Anhydrous, Verapamil, Estrone , Olaparib,Folic Acid,Somatropin, Ibrutinib, Warfarin, Diethylstilbestrol, Thormbon, Melatonin, Calcitriol, Thioguanine, Temozolomide, Sulindac, Troglitazone, Indomethacin, Ndomethacin, Bromocriptine, Vorinostat, Imatinib, Lithium, Nifedipine, Bevacizumab-Awwb, Methyldopa Anhydrous, Curcumin, Ribavirin, Fenofibrate, Metronidazole, Infliximab-Dyyb, Gemfibrozil, Linezolid, Midostaurin, Saquinavir, Nelfinavir, Vitamin K, Echinacea Preparation, Gallium Nitrate, Vitamin A Palmitate, Selenium, Siltuximab, Rituximab, Adalimumab-Adbm, Peginterferon Alfa-2B, Etanercept-Szzs, Ifosfamide Fentanyl Citrate, Levofloxacin Anhydrous, Peginterferon Alfa-2A, Interferon Alfa-2B, Duloxetine Hydrochloride,  Irinotecan Hydrochloride, Crizotinib, Trofinetide, Niclosamide, Acitretin, Digitoxin, Pyrimethamine, Digoxin, Azacitidine, Epoetin Alfa, Bortezomib, Pamidronate, Alprazolam, Naproxen Sodium, Lansoprazole, Danazol, Hydroquinone, Pyrogallol, Vitamin A, Ceftriaxone, Paclitaxel, Cyclophosphamide Anhydrous, Terfenadine, Clarithromycin, Pazopanib, cetaminophen, Medroxyprogesterone Acetate, Ranibizumab, Talc, Midazolam Hydrochloride, Alpha-Tocopherol Acetate, Colchicine, Pentoxifylline, Dacarbazine, Leflunomide, Ibuprofen, Sodium Salt, Cidofovir Anhydrous, Methimazole, Fenretinide, Aluminum Hydroxide, Methylene Blue Anhydrous, Foscarnet, unitinib, Omeprazole |
| Drug bank | Yttrium Y-90, Epirubicin, Chymotrypsin,  Acetylsalicylic acid |
