# Supplementary material for: Exploring common genomic biomarkers to disclose common drugs for the treatment of colorectal cancer and hepatocellular carcinoma with type-2 diabetes through transcriptomics analysis
Source: PLoS One. 2025 Mar 24;20(3):e0319028. doi: 10.1371/journal.pone.0319028 (PMC11932495; doi:10.1371/journal.pone.0319028)
Supplement: S2 Table — (DOCX) [file pone.0319028.s009.docx]

| **S2 Table. Collection of candidate drug agents for HCC from published articles and additional sources.** | |
| --- | --- |
| Articles | Drug agents |
| Liu, Chen, and Chen 2015[24] | Sorafenib, Piperine, curcumin, oleocanthal, tivozanib, 5-FU |
| Peck-Radosavljevic 2014[25] | regorafenib, tivantinib, cabozantinib, refametinib |
| Li et al. 2019[26] | Romidepsin, panobinostat, idarubicin, daunorubicin, topotecan |
| Banerjee and Saluja 2015[27] | Minnelide |
| Feng et al. 2020[28] | Lenvatinib, Donafenib, Apatinib, Milciclib, Palbociclib, Ribociclib, fisogatinib |
| Liang Li and Wang 2016[29] | erlotinib, gefitinib, sunitinib |
| Liang Li and Wang 2016[29] | zebularine |
| Liang Li and Wang 2016[29] | Futibatinib |
| Liang Li and Wang 2016[29] | Infigratinib phosphate. |
| [29] | Pemigatinib. |
|  | Linsitinib. |
| Liang Li and Wang 2016[29] | mitomycin C, gemcitabine, irinotecan,doxorubicin, pirarubicin |
| Liang Li and Wang 2016[29] | oridonin |
| Liang Li and Wang 2016[29] | metformin, Soraphen A, 5-(Tetradecyloxy)-2-furoic acid, zoledronic acid, minodronate, Pamidronic |
| Caruso et al. 2019[30] | Alvespimycin, Trametinib, cd532, Tanespimycin, Dasatinib, Alisertib, MK-2206, Resminostat, Pha-665752, Linsitinib, Entinostat, BLU9931, Selumetinib, Nutlin-3, ICG-001, Decitabine, Brivanib, JNJ-38877605. |
| dgidb n.d , june1st 2024[31] | chembl1082552, aruncin b, protuboxepin a, riviciclib, ag-24322, dinaciclib, rg-547, cinnarizine, rgb-286638,  seliciclib, alvocidib, patulin, chembl403183, withaferin a, zotiraciclib, genistein, tamoxifen, amonafide, chembl607534, chembl594259, ungeremine, chembl507986, lupeol, diazirine, huratoxin, chembl596082,  oleanderolide, tricitrinol b, chembl594257, chembl594153, makaluvamine e, frangulin b, makaluvamine f,  chembl593570, chembl594379, chembl594695, demethylzeylasterone, elinafide, chembl1080077, chembl244268, kaempferitrin, betulin,  4'-o-acetylpatentiflorin b, chembl1773343, amrubicin, dexrazoxane, teniposide, triapine, ly-2334737, cladribine, tezacitabine, fludarabine phosphate, gemcitabine hydrochloride, hydroxyurea, clofarabine, cytarabine, tas-119, mk-5108, pf-03814735, nerviano, danusertib, amg-900, at-9283, norharmane, mk-6592, eupatorin, enmd-2076, kw-2449, mln-8054,  sns-314, dimethyladenine, tozasertib |
