# Supplementary material for: Exploring common genomic biomarkers to disclose common drugs for the treatment of colorectal cancer and hepatocellular carcinoma with type-2 diabetes through transcriptomics analysis
Source: PLoS One. 2025 Mar 24;20(3):e0319028. doi: 10.1371/journal.pone.0319028 (PMC11932495; doi:10.1371/journal.pone.0319028)
Supplement: S3 Table — (DOCX) [file pone.0319028.s010.docx]

| **S3 Table: Collection of candidate drug agents for T2D from published articles and additional sources.** | |
| --- | --- |
| Articles | Drug agents |
| Bailey et al. 2016 [32] | Alogliptin, Saxagliptin, Sitagliptin, Lixisenatide, Empaglifl ozin, Liraglutide, Canaglifl ozin, Exenatide, Linagliptin, Dapaglifl ozin, Dulaglutide, Acarbose |
| Krentz et al. 2008 [33] | Sulfonylureas, Meglitinides, Metformin, Thiazolidinediones, α-Glucosidase, nhibitors, Insulin |
| Esposito et al. 2012 [34] | Basal, Biphasic, Prandial, Basal bolus, GLP-1 agonists, Exenatide LAR, DPP-4 inhibitors, AGI, Thiazolidinediones, Sulphonylureas, Glinides, Metformin |
| Moller et al. 2001 [35] | Insulin, Sulphonylureas, Metformin, Acarbose, Pioglitazone, rosiglitazone |
| Chehade et al. 2000 [36] | Acetohexamide, olbutamide, Chlorpropamide, Tolazamide, Glipizide, Glipizide, Glyburide, Glyburide micronised, Glimepiride, Gliquidone, Gliclazide |
| Deans et al. 2006 [37] | Statins, Fibrates, ACE inhibitors and ARBs, Aspirin, Metformin, Glitazones |
| Avery et al. 2008 [38] | Ciglitazone, Troglitazone, Pioglitazone, Englitazone, Darglitazone, Rosiglitazone, Isaglitazone |
| Paneni et al. 2017 [39] | Biguanides, Sulfonylureas, Thiazolidinediones, peptide-1 receptor, Dipeptidyl peptidase-4, Sodium glucose |
| Adeghate et al. 2021 [40] | Sulfonylureas, Biguanides, Inhibitors of α-glucosidase, Thiazolidinediones, Meglitinides, Dipeptidyl peptidase 4 inhibitors |
| Gupta et al. 2009 [41] | Insulin, Sulphonylurea, Biguanides, Acarbose, Thiazolidinediones, PPAR/ dual agonists, GLP-1 analogs |
| Tamborlane et al. 2016 [42] | Colesevelam, Exenatide, Linagliptin, Liraglutide, Saxagliptin, Sitagliptin, Taspoglutide, Empagliflozin, Exenatide, Alogliptin, Albiglutide, Omarigliptin, Dulaglutide, Lixisenatide, Sotagliflozin, Ertugliflozin |
| Lu et al. 2018 [43] | Insulin and its derivatives, Sulphonylurea, Biguanide, Acarbose, Voglibose, Pioglitazone, Exercise, Curcumin, Resveratol, α-thioctic acids, |
| Thrasher et al. 2017 [44] | Metformin, Albiglutide, Dulaglutide, Exenatide, Exenatide XR, Liraglutide, Lixisenatide, Canagliflozin, Dapagliflozin, Empagliflozin, Alogliptin, Linagliptin, Sitagliptin, Saxagliptin, Glimepiride, Glipizide, Glyburide, Pioglitazone, Rosiglitazone |
| Kalsi et al 2015 [45] | Thiazolidinediones, Biguanide, Sulfonylureas, Meglitinides, Insulin |
| Olokoba et al. 2012 [46] | Biguanides, Sulfonylureas, Meglitinides, Thiazolidinediones, Alpha-Glucosidase Inhibitors, Incretin, Bromocriptine, Insulin |
| Kong et al. 2021 [47] | Berberine, Resveratrol, Emodin, Ellagic acid, Epigallocatechin gallate, Curcumin, Baicalein, Naringenin, Hesperetin, Chrysin, Genistein, Kaempferol, Eriodictyol, Apigenin, Quercetin |
| Tripathi et al. 2013 [48] | Steroids, Triterpenoids, Saponins, Glycosides, Carbohydrates, Alkaloids, Flavonoids, Tannins & Phenolic, Proteins, Amino acid |
| Hassan et al. 2022 [49] | Tannin, Saponin, Alkaloid, Flavonoid, Phenol, Terpenoid, Carbohydrate |
| Yasmin et al. 2020 [50] | Singrin, Boeravinone E, Boeravinone D, Wedelolactone, Squamosamide , Taxifolin |
| Singh et al. 2019 [51] | Polyphenols, Amino Acid, Saponins, Terpenoids, Abscisic Acid, Lycopene and b-Carotene, Oxyphytosterol, Phytosterols/Stanols |
| González-Castejón et al. 2011 [52] | Epigallocatechin gallate, Cyandin and cyanidin 3-glucoside, Soy isoflavones mixture, Genistein, Naringin, Quercetin, Berberine, Resveratrol |
| Hariftyani et al. 2021 [53] | 6-Methoxykaempferol, α-Bisabolol, Anthecotulide, Apigenin, Apigenin-7-glucoside, Axillarin, Azulene, β-Sitosterol, Caffeic acid, Catechin, Chlorogenic acid, Chrysoeriol, Chrysosplenetin, Eupatoletin, Isoferulic acid, Isorhamnetin, Jaceidin, Kaempferol, Linoleic acid, Luteolin, Matricarin, Oleic acid, Patuletin, Quercetagetin-3,6,7,3',4'-, pentamethylether, Quercetin, Sinapic acid, Spinacetin, Stigmasterol, Thiamine, Umbeliferone. |
| Adefegha et al. 2013 [54] | saponins, alkaloids, terpenes, phenylpropanoids, isoprenoids, steroids, coumarins, flavonoids, phenolic acids, lignans, contain chemicals such as flavonoids, terpenoids, lignans, sulfides, polyphenolics, carotenoids, coumarins |
| Atere et al. 2017 [55] | (9Z,12Z)-octadeca-9,12-dienoic acid, 4-[Bis(2-hydroxyethyl)amino]phenyl]-1,1,2-ethylenetricarbonitrile, 1-(2-(methylthio)pyrimidin-4-yl)ethanone, 1-(2-chloropyridin-4-yl)ethanone, 1-(furan-2-yl)ethanone, 1,19-Eicosadiene, 2-methoxyphenol, 2-Pentadecanone, 2-Phenyloxazole-4-carboxylic acid, 2-Propenoic acid-3-(4-hydroxy-3-methethoxyphenly ferullic acid, 2,3-Dihydrobenzofuran-5-boronic acid, 2,6-Dimethoxyphenol, 7-decen-4-olide, 14-Methylpentadecanoic acid, Citronellyl isobutyrate, E stilbene, Inositol 1,4,5-trisphosphate, Methyl stearate, n-Hexadecanoic acid, Phenol, 2,6-bis(bromomethyl)-4-chloro-3,5-dimethyl, Phytol |
| Rampogu et al. 2018 [56] | Flavanoid, Tannin, Terpenoids, Alkaloids, Saponins |
| Kausar et al. 2021 [57] | 4,5-dimethyl-3-hydroxy-2(5H)-furanone, apigenin, bromelain, caffeic acid, cholecalciferol, dihydrokaempferol 7-o-glucopyranoside, galactomannan, genkwanin, isoimperatorin, luteolin, luteolin 7-o-glucoside, neohesperidin, oleanoic acid, pelargonidin-3-rutinoside, quercetin, quinic acid |
| Sari et al. 2021 [58] | 2,3-Butanediol, Glycerin, 4H-Pyran-4-one,2,3- dihydro-3,5-dihydroxy6-methyl, 5- Hydroxymethylfurfural, D-Glucose,6-O-à-Dgalactopyranosyl, Sucrose, Cyclooctasiloxane, hexadecamethyl -, n -Hexadecanoic acid, 4H -Pyran - 4 -one,5 - hydroxy - 2 – (hydroxymethyl) |
| Onaolapo et al. 2018 [59] | Flavonoids, Alkaloids, Glycosides, Polysaccharides, Terpenoids and steroids, Saponins Isothiocyanate, |
| Belobrajdic et al. 2013 [60] | Methionine, Cystine, Selenium, Folate, Choline, Tocopherols + tocotrienols, Carotenoids, Polyphenols, Phenolic acids, Phenolic acid, Ferulic acid, Flavanoids, Alkylresorcinols, Avenanthramides, Betaine, Phytosterols |
| Oboh et al. 2014[61] | Coumarin, p-Coumaric acid, o-Coumaric acid, Vanillic acid, Caffeic acid, Ferulic acid, Syringic acid, Sinapinic acid, Genistein, Apigenin, Naringenin, Kaempferol, Luteolin, Epicatechin, Epigallocatechin, Quercetin, 2-Phenylethyl-β-D-glucoside, Phenyl-6I-O-maloyl-β-D-glucoside |
