# Supplementary material for: Exploring common genomic biomarkers to disclose common drugs for the treatment of colorectal cancer and hepatocellular carcinoma with type-2 diabetes through transcriptomics analysis
Source: PLoS One. 2025 Mar 24;20(3):e0319028. doi: 10.1371/journal.pone.0319028 (PMC11932495; doi:10.1371/journal.pone.0319028)
Supplement: S4 Table — (DOCX) [file pone.0319028.s011.docx]

| **S4 Table: Collection of Colorectal Cancer (CRC) causing KGs from different published articles to select top-ranked publicly available receptors** | | |
| --- | --- | --- |
| **Article** | **Key genes** |  |
| [62] | TIMP1, SLC4A4, AKR1B10, ABCE1 | Common genes at least 3 articles  TIMP1, TOP2A, MAD2L1, CXCL12, AURKA, CDK1, PYY, SST, CXCL1, MYC, LPAR1, NMU |
| [63] | TOP2A, MAD2L1, CDC6, CHEK1 |  |
| [64] | ACTA2, ACTG2, MYH11, CALD1, MYL9, TPM2, and LMOD1 |  |
| [65] | APC, KRAS, BRAF, PIK3CA, SMAD4 and p53 |  |
| [66] | AHSG, SERPINC1, FGA, F2, CP, ITIH2, APOA2, HPX, PLG, HRG, TIMP1, CXCL1, COL1A2, MMP1, AURKA, UBE2C, CXCL12, TOP2A, ALDH1A1 and PRKACB |  |
| [67] | CDK1, CEP55, MKI67, and TOP2A |  |
| [68] | AURKA, CCNA2, CCNB1, CDK1, CKS2, DLGAP5, and MAD2L1 |  |
| [69] | CDK1, CCNB1, MAD2L1, BUB1B, SOX4, COL1A2 and MYC |  |
| [70] | AURKA, TOP2A, CDK1, PTTG1, CDKN3, CDC20, MAD2L1, CKS2, MELK, and TPX2 |  |
| [71] | CCL19, CXCL1, CXCL5, CXCL11, CXCL12, GNG4, INSL5, NMU, PYY, and SST |  |
| [72] | MYC, CXCL1, CD44, MMP1, and CXCL12 |  |
| [73] | IL8, CXCL1, CXCL10, CCL20, CCL21, GRM8, CXCL12, SST, PYY and LPAR1 |  |
| [74] | CXCL1, CXCL3, CXCL8, CXCL11, NMU, and PPBP |  |
| [75] | BIRC5, CCNB1, KIF20A, NCAPG, and TPX2 |  |
| [76] | GNG4, PPBP, NMU, LPAR1, CXCL1, HIST1H2BJ, CXCL8, PYY, HIST1H2AD and SST |  |
| [77] | COL1A1, CXCL5, GNG4, TIMP1, SPP1, and LPAR1 |  |
| [78] | COL1A2, THBS2, TIMP1, and CXCL8 |  |
| [79] | ZG16, TIMP1, and BGN |  |
| [80] | AURKA, CCNB1, CCNF, and EXO1 |  |
| [81] | SST, PYY, GNG12, CCL13, MCHR2, CCL28, ADCY9, SSTR1, CXCL12 and ADRA2A |  |
| [82] | AURKA, CCNB1, EXO1 and CCNA2 |  |
| [83] | IGF1, MYH11, CLU, MYL9, CXCL12, LMOD1, C3, CNN1, FOS, and HIST1H2BO |  |
| [84] | EPHB2, KLK8, DIAPH3, STC2, OXTR, MMP7, MET, KRT85, KRT6B, KRT23, and KLK10 |  |
| [85] | IL6, MYC, NOTCH1, INHBA, CDK1, cyclin (CCN)B1 and CCNA2 |  |
| [86] | CCND1, MYC, PIK3R2 and SMAD3 |  |
| [87] | ALB, F2, APOH, SERPINC1, APOA1, AMBP, APOC3, PLG, AHSG and APOB |  |
| [88] | SPP1, VIP, COL11A1, CA2, ADAM12, INHBA |  |
| [89] | CEACAM7, SLC4A4, GCG and CLCA1 |  |
| [90] | PPBP, CCL28, CXCL12, INSL5, CXCL3, CXCL10, and CXCL11 |  |
| [91] | FOS, FN1, PPP1CC, and CYP2B6 |  |
