# Supplementary material for: Exploring common genomic biomarkers to disclose common drugs for the treatment of colorectal cancer and hepatocellular carcinoma with type-2 diabetes through transcriptomics analysis
Source: PLoS One. 2025 Mar 24;20(3):e0319028. doi: 10.1371/journal.pone.0319028 (PMC11932495; doi:10.1371/journal.pone.0319028)
Supplement: S5 Table — (DOCX) [file pone.0319028.s012.docx]

| **S5 Table. Collection of Hepatocellular carcinoma (HCC) causing KGs from different published articles to select top-ranked publicly available receptors** | | |
| --- | --- | --- |
| **Articles** | **Key genes** | **Repeated key genes** |
| [92] | CDC20, CCNB1, EIF4A3, H2AFX, NOP56, RFC4, NOP58, AURKA, PCNA, and FEN1 | Common genes at least 4 articles:  AURKA  CCNB2  CCNB1  CDK1  CDC20  TOP2A  RFC4  BUB1  PRC1  RRM2 |
| [93] | CDKN3, CDK1, CCNB1, TOP2A, CCNA2, CCNB2, PRC1 |  |
| [94] | CCNB1, CDC20, and CENPF |  |
| [95] | CDK1, CCNB1, CCNB2, MAD2L1, and TOP2A |  |
| [96] | CDK1, CCNB1, CDC20, BUB1, MAD2L1, MCM3, BUB1, MCM2, and RFC4 |  |
| [97] | CCNB2, TOP2A, and ASPM |  |
| [98] | MAD2L1, CDC20, CCNB1, CCND1, AURKA and ESR1 |  |
| [99] | CDK1, NDC80, HMMR, CDKN3, and PTTG1 |  |
| [100] | TTK, NCAPG, TOP2A, CCNB1, CDK1, PRC1, RRM2, UBE2C, ZWINT, CDKN3, AURKA, and RACGAP1 |  |
| [101] | TOP2A, PCNA, CCNB2, AURKA, CDKN3, BUB1, RFC4, CEP55, DLGAP5, MCM2, PRC1, RACGAP1, TPX2, CDC20, and MCM4. |  |
| [102] | PCNA, RFC4, PTTG1, H2AFZ, and RRM1 |  |
| [103] | BUB1 |  |
| [104] | CCNB2, CDC20, AURKA, TOP2A, MELK, NCAPG, KIF20A, UBE2C, PRC1 |  |
| [105] | TOP2A, CDC20, MAD2L1, BUB1B, RFC4, CCNB1, CDKN3, CCNB2, TPX2, and FEN1 |  |
| [106] | CDC20, CCNB2, NCAPG, ASPM and NUSAP1 |  |
| [107] | RRM2, MAD2L1, MELK, NCAPG, and ASPM |  |
| [108] | RRM2, NDC80, ECT2, CCNB1, CDK1, PRC1, KIF20A, DTL, TOP2A, and PBK |  |
| [109] | ANLN, BUB1B, CCNB1, CDK1, CDKN3, ECT2, HMMR, NEK2, PBK, PRC1, RACGAP1, RRM2, and TOP2A |  |
| [110] | CDKN3, TOP2A, UBE2C, CDC20, PBK, KIF20A, NCAPG, CCNB2, CYP3A4. |  |
| [111] | KIF20A, HMMR, RRM2, TPX2, TTK and UBE2C |  |
