# Supplementary material for: Exploring common genomic biomarkers to disclose common drugs for the treatment of colorectal cancer and hepatocellular carcinoma with type-2 diabetes through transcriptomics analysis
Source: PLoS One. 2025 Mar 24;20(3):e0319028. doi: 10.1371/journal.pone.0319028 (PMC11932495; doi:10.1371/journal.pone.0319028)
Supplement: S6 Table — (DOCX) [file pone.0319028.s013.docx]

| **S6 Table: Collection of Type-2 diabetes (T2D) causing KGs from different published articles to select top-ranked publicly available receptors** | | |
| --- | --- | --- |
| **Articles** | **Key genes** | **Repeated key genes** |
| [112] | IL6, MMP3, INHBA, MMP1, VCAN, FGF7, PDGFRA, THBS2, IL11, CYR61 | Common genes at least 3 articles:  IL6  LUM  C3AR1  PTP1B  HHEX  ATF3  ALB  KCNJ1  AMPK  EGF |
| [113] | ACLY, SLC2A2, ARHGEF9, EDN3, RRAGD, MAK16, RHOBTB3, HADH, TRIM37, CLGN, CTNNA2, ACLY, ABHD10, ATRNL1, ENTPD3, GRAMD3, CCL21, TGFBR3, NBEA, IAPP, APOBEC2, FKBP2, GRHL2, ITGA3, PID1, PTGES, S100A14, S100A6, SAMSN1 and THBS2 |  |
| [114] | MYH9, FLNA, DCTN1, CLTC, ERBB2, TCF4, VIM, LRRK2, IFI16, CAV1 |  |
| [115] | JUN, VCAM1, RELA, U2AF2, ADRB2, FN1, CDK1, TK1, A2M and ACTA2 |  |
| [116] | SERPING1, ANPEP, HNF1A, STAT3, GR, ACE2 |  |
| [117] | CEBPD, TP73, ESR2, TAB1, MAP ,3K5, FN1, UBD, RUNX1, PIK3R2, TNF |  |
| [118] | KCNJ11, TCF7L2, PPARG, CDKL1, HHEX, NOTCH, FTO, TCF7L2, NOTCH2, WFS1, CDKAL1 IGF2BP2, SLC30A8, JAZF1, HHEX, SUZ12, EZH2, ZNF580, KLF9, MAZ, ATF1, SSRP1, WRNIP1, CHD1, PRKCB, SP1, GRB14, LYN, ITGA5, PIK3R1, RAC1, GNG3, GNAI1, CDC42, ITGB1, IL8, FPR1, CXCL1, GNAI3, FPR2, GNAI2, ANXA1, GNB1, LPAR5 |  |
| [119] | CD36, GLUL, COL4A2, ACACB |  |
| [120] | CDNF, CRELD2, DNAJB11, DTL, GINS2, MANF, PDIA4, PDIA6, VCP |  |
| [121] | CNOT6L, CNOT6, CNOT1, CNOT7, RQCD1, RFC2, PRIM1, RFC4, RFC5, RFC1 |  |
|  | CCL2, ELMO1, VEGFA, TCF7L2 |  |
| [122] | NSF, ADD2 |  |
| [32] | RBP4, AMPK |  |
| [33] | AMPK |  |
| [34] | HbA1c |  |
| [123] | PTP-1B, SHIP-2, GSK-3, IkB kinase |  |
| [124] | ACE |  |
| [38] | PPAR, AMPK, PTP1B |  |
| [41] | DPP IV, GLP-1, GIP, PPARalpha, PPARgamma |  |
| [44] | GLP-1, HbA1c, DPP-4, SGLT2 |  |
| [45] | AMPK, PPARγ, GLUT4 |  |
| [46] | GLP-1, GIP, DPP-IV, TNF-α, HbA1c, TCF7L2, PPARG, FTO, KCNJ11, NOTCH2, WFS1, CDKAL1, IGF2BP2, SLC30A8, |  |
| [47] | JAZF1, and HHEX. KCNJ11, IL-6, IL-1β, TNF-α, AMPK, PPAR-γ, PTP-1B |  |
| [51] | PKC, MAP, GFAP, HSA, AMPK, GLP-1, DPP-4 |  |
| [52] | PPAR, PPAR, IL-6, MCP-1 and IL-1, TNF |  |
| [53] | PTP1B[, AKR1B1](https://www.rcsb.org/search?q=rcsb_entity_source_organism.rcsb_gene_name.value:AKR1B1) |  |
| [54] | TGF-β, AGEs, IL-8 |  |
| [55] | DPP-IV, PTP-1β, PPAR- γ, GIP, GIP-1 |  |
| [56] | PTP1B |  |
| [57] | AGS |  |
| [58] | SGLT2 |  |
