# Supplementary material for: Exploring common genomic biomarkers to disclose common drugs for the treatment of colorectal cancer and hepatocellular carcinoma with type-2 diabetes through transcriptomics analysis
Source: PLoS One. 2025 Mar 24;20(3):e0319028. doi: 10.1371/journal.pone.0319028 (PMC11932495; doi:10.1371/journal.pone.0319028)
Supplement: S7 Table — (DOCX) [file pone.0319028.s014.docx]

| **S7 Table: List of cDEGs, including upregulated and downregulated genes, among CRC, HCC, and T2D.** | |
| --- | --- |
| **Downregulated sDEGs** | **Upregulated sDEGs** |
| ADH1C, AHNAK, ANPEP, C4orf19, CCDC68, CFB, CITED2, CYP3A4, CYP3A5, DEFB1, DUSP5, EDNRB, EP300-AS1, EXPH5, EZR, GIPC2, GK, GNG12, GSTA1, HMOX1, HSD17B2, ID2, IL6ST, KLF4, KLF6, KRT7, MCL1, MGLL, MT1M, NEDD9, NR5A2, PIGR, POU2AF1, PRRG4, PSD3, PTP4A1, RAB27A, SLAMF7, SLC1A1, SLC25A37, SLC41A2, SLC4A4, STS, TGFA, CXCL16, TLR3, TPBG, UGT2A3, UGT2B15 | ACKR3, APOLD1, AREG, BIRC5, CCL20, CDK6, CKS2, CLDN1, CLDN2, CXCL1, CXCL10, THBS1, CXCL2, CXCL3, CXCL5, CXCL6, CXCL8, CXCL9, DUSP10 , DUSP4, ETS2, GDF15, GNG4, GRK3, ICAM1 IL1RN, IL6, IRS1, ITGA2, LCN2, LDLR, MMP9, MYC, PMAIP1, RRM2, SPP1, TNFSF15 |
