# Supplementary material for: Exploring common genomic biomarkers to disclose common drugs for the treatment of colorectal cancer and hepatocellular carcinoma with type-2 diabetes through transcriptomics analysis
Source: PLoS One. 2025 Mar 24;20(3):e0319028. doi: 10.1371/journal.pone.0319028 (PMC11932495; doi:10.1371/journal.pone.0319028)
Supplement: S8 Table — (DOCX) [file pone.0319028.s015.docx]

| **S8 Table: aLog*_2_*FC values of cDEGS in T2D, HCC and CRC** | | | | | | | |
| --- | --- | --- | --- | --- | --- | --- | --- |
| Gene | aLog*_2_*FC  (T2D) | aLog*_2_*FC  (HCC) | aLog*_2_*FC  (CRC) | Gene | aLog*_2_*FC  (T2D) | aLog*_2_*FC  (HCC) | aLog*_2_*FC  (CRC) |
| ACKR3 | -1.08562 | -1.14489 | -3.2828 | CXCL5 | 1.867172 | 1.628845 | 2.016818 |
| ADH1C | -1.51204 | -1.84401 | -2.86423 | CXCL6 | 1.553974 | 1.174303 | 4.224022 |
| AHNAK | -1.69772 | -1.50022 | -1.81564 | CXCL8 | 2.048993 | 2.607298 | 3.535654 |
| ANPEP | -2.84846 | -1.63142 | -2.72816 | CXCL9 | 1.587333 | 1.694105 | 1.405228 |
| APOLD1 | -1.86594 | -1.71325 | -2.85642 | CYP3A4 | -1.54237 | -1.93004 | -1.74874 |
| AREG | 1.882066 | 5.497588 | 2.951696 | CYP3A5 | -1.80141 | -1.80015 | -1.02827 |
| BIRC5 | 1.72496 | 3.949553 | 1.545488 | DEFB1 | -1.71978 | -1.24796 | -3.98907 |
| C4orf19 | -1.66617 | -1.7482 | -2.10957 | DUSP10 | 3.509689 | 1.71116 | 1.424658 |
| CCDC68 | -1.54214 | -1.43214 | -2.94351 | DUSP4 | 1.804327 | 1.829844 | 1.998848 |
| CCL20 | 1.695463 | 2.477994 | 2.30814 | DUSP5 | -1.54827 | -1.03656 | -1.59093 |
| CDK6 | 1.80025 | 1.8549 | 1.856455 | EDNRB | -1.85844 | -1.17708 | -1.54849 |
| CFB | -1.17908 | -1.61356 | -1.61064 | EP300-AS1 | -1.7732 | -3.11756 | -1.54808 |
| CITED2 | -1.56067 | -1.55067 | -1.57067 | ETS2 | 1.854334 | 1.994045 | 1.152274 |
| CKS2 | 1.88338 | 1.286869 | 1.71651 | EXPH5 | -1.21153 | -2.43255 | -3.07793 |
| CLDN1 | 3.422599 | 1.205063 | 3.354752 | EZR | -1.69202 | -4.51369 | -4.89781 |
| CLDN2 | 1.060312 | 1.942397 | 2.118134 | GDF15 | 1.99411 | 2.590809 | 3.273054 |
| CXCL1 | 1.592279 | 1.75488 | 3.516655 | GIPC2 | -1.53865 | -1.05222 | -1.99022 |
| CXCL10 | 3.506527 | 1.775521 | 1.523427 | GK | -1.87248 | -1.67489 | -1.82299 |
| CXCL16 | -1.57012 | -1.5292 | -1.39944 | MCL1 | -1.86088 | -3.02944 | -1.09034 |
| CXCL2 | 1.916836 | 4.698673 | 1.059467 | MGLL | -1.58162 | -1.76758 | -1.52972 |
| CXCL3 | 1.548884 | 2.030636 | 2.727943 | MMP9 | 1.572839 | 1.818056 | 1.709697 |
| GNG12 | -1.61306 | -1.97411 | -1.27572 | MT1M | -1.36711 | -2.65166 | -4.79513 |
| GNG4 | 1.70314 | 4.598192 | 1.562012 | MYC | 1.63564 | 2.8494 | 2.542473 |
| GRK3 | 1.688768 | 5.66489 | 1.352568 | NEDD9 | -4.67292 | -1.69027 | -1.43524 |
| GSTA1 | -1.1228 | -2.13579 | -1.90677 | NR5A2 | -1.70769 | -1.51201 | -2.74269 |
| HMOX1 | -3.02025 | -1.59214 | -1.71789 | PIGR | -1.96747 | -1.42741 | -2.55188 |
| HSD17B2 | -1.68744 | -1.25406 | -4.85781 | PMAIP1 | 1.068504 | 1.586054 | 2.35482 |
| ICAM1 | 2.766276 | 1.721779 | 1.257422 | POU2AF1 | -1.99673 | -1.20981 | -2.08926 |
| ID2 | -1.66428 | -3.08986 | -1.75426 | PRRG4 | -1.52718 | -1.76443 | -1.11928 |
| IL1RN | 1.627355 | 1.820681 | 1.087032 | PSD3 | -1.51574 | -1.56176 | -1.12879 |
| IL6 | 1.823269 | 1.536238 | 1.860467 | PTP4A1 | -1.64367 | -1.72566 | -1.3625 |
| IL6ST | -1.56565 | -1.85651 | -1.81151 | RAB27A | -1.58473 | -1.5462 | -1.59612 |
| IRS1 | 1.603766 | 1.678705 | 1.770511 | RRM2 | 1.622192 | 2.761923 | 1.589094 |
| ITGA2 | -1.1998 | -1.72817 | -1.20788 | SLAMF7 | -1.52628 | -1.68725 | -1.44171 |
| KLF4 | -1.50331 | -1.18517 | -2.8821 | SLC1A1 | -1.71847 | -1.94038 | -2.49762 |
| KLF6 | -1.62614 | -1.59946 | -1.69159 | SLC25A37 | -1.52946 | -1.76862 | -1.62079 |
| KRT7 | -1.32641 | -1.6861 | -1.71941 | SLC41A2 | -1.61661 | -1.09873 | -1.99744 |
| LCN2 | 1.588055 | 4.661409 | 2.042162 | SLC4A4 | -1.98095 | -1.18573 | -5.35245 |
| LDLR | 1.682374 | 1.051353 | 1.501001 | SPP1 | 1.591326 | 2.498915 | 2.530362 |
| THBS1 | 1.875679 | 1.256037 | 3.597666 | STS | -1.71972 | -1.66294 | -1.76414 |
| TLR3 | -1.57373 | -1.75631 | -2.52341 | TGFA | -1.47386 | -1.82691 | -1.65922 |
| TNFSF15 | 1.804994 | 1.66794 | 1.3395 | UGT2A3 | -1.55934 | -1.74392 | -4.24817 |
| TPBG | 1.54884 | 1.560169 | 1.760772 | UGT2B15 | -1.78269 | -3.75939 | -1.54331 |
