# Supplementary material for: Exploring common genomic biomarkers to disclose common drugs for the treatment of colorectal cancer and hepatocellular carcinoma with type-2 diabetes through transcriptomics analysis
Source: PLoS One. 2025 Mar 24;20(3):e0319028. doi: 10.1371/journal.pone.0319028 (PMC11932495; doi:10.1371/journal.pone.0319028)
Supplement: S9 Table — (DOCX) [file pone.0319028.s016.docx]

| **S9 Table: List of cGBs from PPI network based on different topological measures** | | | | | | | | |
| --- | --- | --- | --- | --- | --- | --- | --- | --- |
| **SN.** | CGBs | Degree | Closeness | EPC | MNC | Betweenness | Radiality | Stress |
| **1** | MYC | 62 | 72.67 | 19.34 | 62 | 488.3563 | 4.78 | 3722 |
| **2** | MMP9 | 57 | 70.167 | 19.35 | 57 | 227.526 | 4.72 | 2548 |
| **3** | IL6 | 67 | 75.167 | 19.75 | 67 | 812.44 | 4.84 | 5014 |
| **4** | CXCL1 | 49 | 66 | 18.63 | 49 | 179.307 | 4.61 | 1980 |
| **5** | SPP1 | 47 | 64.83 | 18.142 | 46 | 251.6637 | 4.58 | 2152 |
| **6** | THBS1 | 52 | 67.83 | 18.146 | 52 | 332.9358 | 4.67 | 2822 |
