# Supplementary material for: Exploring common genomic biomarkers to disclose common drugs for the treatment of colorectal cancer and hepatocellular carcinoma with type-2 diabetes through transcriptomics analysis
Source: PLoS One. 2025 Mar 24;20(3):e0319028. doi: 10.1371/journal.pone.0319028 (PMC11932495; doi:10.1371/journal.pone.0319028)
Supplement: S10 Table — (DOCX) [file pone.0319028.s017.docx]

| **S10 Table: Association of cGBs with various diseases** | | | | |
| --- | --- | --- | --- | --- |
| **Disease Name** | **Annotation ID** | **Number of cGBs** | **Adj p-value** | **Associated cGBs** |
| Neoplasm Metastasis | C0027627 | 4 | 0.000116 | MYC, SPP1, MMP9, IL6 |
| **Alloxan Diabetes** | C0002152 | 2 | 0.000219 | MMP9, IL6 |
| Chronic Airflow Obstruction | C1527303 | 3 | 0.000293 | CXCL1, MMP9, IL6 |
| **Liver carcinoma** | C2239176 | 3 | 0.000479 | MYC, MMP9, IL6 |
| Pulmonary Fibrosis | C0034069 | 3 | 0.000479 | SPP1, MMP9, IL6 |
| **Streptozotocin Diabetes** | C0038433 | 2 | 0.000603 | SPP1, MMP9, IL6, THBS1 |
| **Diabetes Mellitus, Experimental** | C0011853 | 2 | 0.000677 | SPP1, MMP9, IL6, THBS1 |
| Shock, Hemorrhagic | C0036982 | 2 | 0.000677 | CXCL1, IL6 |
| Alveolitis, Fibrosing | C4721507 | 3 | 0.000677 | SPP1, MMP9, IL6 |
| Premature Birth | C0151526 | 2 | 0.000677 | MMP9, IL6 |
| **Liver Cirrhosis** | C0023890 | 3 | 0.000677 | SPP1, IL6, THBS1 |
| Experimental Hepatoma | C0086404 | 3 | 0.000677 | MYC, SPP1, IL6 |
| Hepatoma, Novikoff | C0019208 | 3 | 0.000718 | MYC, SPP1, IL6 |
| Fibrosis, Liver | C0239946 | 3 | 0.000718 | SPP1, IL6, THBS1 |
| Hepatoma, Morris | C0019207 | 3 | 0.000718 | MYC, SPP1, IL6 |
| **Liver Neoplasms, Experimental** | C0023904 | 3 | 0.000718 | MYC, SPP1, IL6 |
| **Chemically Induced Liver Toxicity** | C4279912 | 4 | 0.000744 | SPP1, CXCL1, MMP9, IL6 |
| **Colonic Neoplasms** | C0009375 | 2 | 0.000808 | MYC, MMP9 |
| **Malignant tumor of colon** | C0007102 | 2 | 0.0018775 | MYC, MMP9 |
| Squamous cell carcinoma | C0007137 | 3 | 0.009904 | MYC, MMP9, IL6 |
| **Drug-Induced Acute Liver Injury** | C3658290 | 4 | 0.009904 | SPP1, CXCL1, MMP9, IL6 |
| **Drug-Induced Liver Disease** | C0860207 | 4 | 0.009904 | SPP1, CXCL1, MMP9, IL6 |
| Autoimmune Chronic Hepatitis | C0241910 | 2 | 0.01082 | CXCL1, IL6 |
| Oral Submucous Fibrosis | C0029172 | 2 | 0.011848 | MMP9, IL6 |
| **Diabetes Mellitus, Insulin-Dependent** | C0011854 | 1 | 0.012102 | IL6 |
