# Supplementary material for: Exploring common genomic biomarkers to disclose common drugs for the treatment of colorectal cancer and hepatocellular carcinoma with type-2 diabetes through transcriptomics analysis
Source: PLoS One. 2025 Mar 24;20(3):e0319028. doi: 10.1371/journal.pone.0319028 (PMC11932495; doi:10.1371/journal.pone.0319028)
Supplement: S11 Table — (DOCX) [file pone.0319028.s018.docx]

| **S11 Table: Performance scores of the Random Forest-based prediction model.** | | | | | | |
| --- | --- | --- | --- | --- | --- | --- |
|  | **Hepatocellular carcinoma (HCC)** | | **Colorectal**  **Cancer (CRC)** | | **Type-2 Diabetes**  **(T2D)** | |
|  | **Train Data**  **(60%)** | **Test Data**  **(40%)** | **Train Data**  **(60%)** | **Test Data**  **(40%)** | **Train Data**  **(60%)** | **Test Data**  **(40%)** |
| **Area Under the ROC Curve (AUC)** | 0.99 | 0.94 | 1 | 0.96 | 0.99 | 0.93 |
| **Accuracy (ACC)** | 0.88 | 0.820 | 0.98 | 0.9375 | 0.98 | 0.90 |
| **True Positive Rate (TPR)** | 1 | 0.88 | 1 | 0.90625 | 0.97 | 0.86 |
| **True Negative Rate (TNR)** | 1 | 0.97 | 0.84 | 0.96875 | 0.82 | 0.94 |
| **False Negative Rate (FNR)** | 0.03 | 0.056 | 0.07 | 0.09 | 0.02 | 0.07 |
| **False Positive Rate (FPR)** | 0.0333 | 0.021 | 0.0333 | 0.03125 | 0.03 | 0.05 |
| **False Discovery Rate (FDR)** | 0.0415 | 0.046 | 0.03225 | 0.0333 | 0.03 | 0.05 |
