# Supplementary material for: Exploring common genomic biomarkers to disclose common drugs for the treatment of colorectal cancer and hepatocellular carcinoma with type-2 diabetes through transcriptomics analysis
Source: PLoS One. 2025 Mar 24;20(3):e0319028. doi: 10.1371/journal.pone.0319028 (PMC11932495; doi:10.1371/journal.pone.0319028)
Supplement: S12 Table — (DOCX) [file pone.0319028.s019.docx]

| **S12 Table: Docking (binding affinity) scores (kcal/mol) between the proposed target genes/proteins (receptors) and the top-ranked candidate drugs.** | | | | | | | | |
| --- | --- | --- | --- | --- | --- | --- | --- | --- |
| Drug | MMP9 | SPP1 | IL6 | THBS1 | FOXC1 | GATA2 | CXCL1 | MYC |
| Digitoxin | -11.5 | -10.3 | -8.6 | -9.8 | -9.2 | -8.1 | -9.1 | -7.7 |
| AMG_900 | -11.3 | -10.2 | -8.3 | -8.8 | -8.1 | -8 | -7.8 | -7.6 |
| Imatinib | -11.2 | -10 | -9.5 | -9.1 | -8.3 | -7.8 | -7.4 | -7.2 |
| Camptosar | -11.5 | -9.4 | -8 | -7.9 | -8.1 | -8 | -7.2 | -7.1 |
| irinotecan | -10.5 | -9.9 | -7.8 | -8.4 | -8.2 | -7.2 | -7.8 | -7.1 |
| Linsitinib | -10.5 | -9.5 | -7.8 | -8.4 | -8.4 | -7.1 | -7.8 | -7.3 |
| Midostaurin | -8.5 | -9.4 | -8.4 | -7.1 | -7.5 | -7.6 | -7.6 | -7.2 |
| INC_280 | -12.2 | -9 | -8.3 | -7.9 | -7.9 | -6.8 | -7.4 | -6.4 |
| Olaparib | -11.4 | -10 | -8.2 | -7.7 | -7.5 | -7 | -7.7 | -6.2 |
| JNJ_38877605 | -11 | -9.6 | -8 | -8.1 | -8.3 | -7.3 | -7.1 | -6.3 |
| GLP_1 | -11.7 | -9.2 | -7.7 | -8.2 | -7.5 | -7.3 | -7.6 | -6.4 |
| ICG_001 | -11.7 | -9.5 | -7.7 | -7.9 | -7.4 | -7.7 | -7.3 | -6.2 |
| Tivantinib | -9.8 | -10.3 | -8.3 | -8.3 | -7.8 | -6.6 | -8 | -6.1 |
| tivantinib | -10.3 | -9.4 | -9.1 | -7.4 | -7.8 | -7.2 | -7.5 | -6.3 |
| Isaglitazone | -10.3 | -9.2 | -9 | -6.8 | -8.6 | -7.1 | -7.5 | -6.2 |
| regorafenib | -11.6 | -9.5 | -7.7 | -7.3 | -7.6 | -8 | -6.7 | -6.2 |
| Taraxerone | -10.7 | -8.9 | -7.6 | -8.3 | -7.6 | -8 | -7.1 | -6.3 |
| Herbacetin | -9.4 | -10 | -7.7 | -8.2 | -8.3 | -6.7 | -7.7 | -6.5 |
| Gliquidone | -10.6 | -9.7 | -7.7 | -8.1 | -7.7 | -7.5 | -7.1 | -5.9 |
| Donafenib | -9.2 | -9.2 | -8 | -7.5 | -8 | -8.3 | -7.2 | -6.8 |
| cabozantinib | -10.5 | -8.8 | -7.8 | -8.1 | -7.6 | -7.8 | -7.3 | -6.3 |
| Oleanderolide | -11 | -9 | -8.1 | -7.6 | -7.2 | -7.9 | -7 | -6.1 |
| Friedelin | -8.6 | -9.2 | -7.9 | -8.3 | -8.7 | -7.2 | -7.4 | -6.4 |
| RGB_286638 | -9.3 | -9.7 | -7.9 | -7.8 | -8.4 | -6.6 | -7.9 | -6 |
| entrectinib | -10.5 | -8.4 | -8.3 | -8.2 | -7.8 | -7.3 | -7 | -6 |
| b_Carotene | -10.9 | -8.8 | -7.8 | -7 | -8 | -7.8 | -7.2 | -5.9 |
| Glimepiride | -9.9 | -9.6 | -8.2 | -7.3 | -7.2 | -7 | -7.3 | -6.8 |
| Sorafenib | -10.5 | -8.9 | -7.8 | -7.6 | -7.1 | -7.6 | -7.4 | -6.2 |
| Diosmin | -10.5 | -9.3 | -7.6 | -7.8 | -7.4 | -7.5 | -6.7 | -6.3 |
| PF_03814735 | -11.7 | -9.5 | -7.1 | -8 | -6.9 | -6.6 | -7.2 | -5.8 |
| Bromocriptine | -10.9 | -9.2 | -7.2 | -7.9 | -7.5 | -7.2 | -7.1 | -5.8 |
| Taraxasterol | -8.9 | -9.9 | -8 | -7.7 | -7.3 | -7.6 | -7 | -6.3 |
| AG_24322 | -8.6 | -9.5 | -8.4 | -8.3 | -8.2 | -6.9 | -7 | -5.7 |
| MK_6592 | -11 | -9.3 | -8.2 | -6.9 | -7.4 | -6.5 | -7.1 | -6.2 |
| Acacetin | -10.8 | -8.6 | -7.9 | -7.6 | -7.4 | -6.7 | -7.2 | -6.4 |
| TENIPOSIDE | -12.2 | -8.4 | -7.1 | -7.6 | -7.6 | -7.7 | -6.7 | -5.3 |
| Darglitazone | -11.3 | -8.2 | -7.6 | -7.5 | -7.9 | -7 | -7.3 | -5.6 |
| Tinyatoxin | -10.4 | -9 | -7.6 | -7.4 | -6.9 | -7.7 | -7.3 | -6 |
| Midostaurin | -10.9 | -9.1 | -7.7 | -6.8 | -7.3 | -7.6 | -6.4 | -6.5 |
| Hesperidin | -8.5 | -9.4 | -8.4 | -7 | -7.5 | -7.6 | -7.6 | -6.2 |
