# Supplementary material for: Exploring common genomic biomarkers to disclose common drugs for the treatment of colorectal cancer and hepatocellular carcinoma with type-2 diabetes through transcriptomics analysis
Source: PLoS One. 2025 Mar 24;20(3):e0319028. doi: 10.1371/journal.pone.0319028 (PMC11932495; doi:10.1371/journal.pone.0319028)
Supplement: S13 Table — (DOCX) [file pone.0319028.s020.docx]

| **S13 Table: Docking (binding affinity) scores (kcal/mol) between the published target genes/proteins (receptors) and the top 7 proposed drugs.** | | | | | | | | |
| --- | --- | --- | --- | --- | --- | --- | --- | --- |
| CRC | Drug | Digitoxin | Camptosar | AMG_900 | irinotecan | Imatinib | Midostaurin | Linsitinib |
|  | AURKA | -11.7 | -10.3 | -10.1 | -9.7 | -9.1 | -10.8 | -9.3 |
|  | CDK1 | -10.9 | -10.3 | -10.2 | -10.2 | -9.2 | -9.3 | -9.1 |
|  | TOP2A | -10.2 | -9.1 | -9.9 | -9.1 | -9.1 | -8.2 | -8.7 |
|  | TIMP1 | -8.5 | -9.2 | -9 | -9.4 | -9 | -8.1 | -8.5 |
|  | MAD2L1 | -9.9 | -8.4 | -9 | -8.5 | -8.6 | -8.4 | -7.9 |
|  | CXCL12 | -8.9 | -8.5 | -7.5 | -8 | -8.1 | -6.9 | -7.4 |
|  | CXCL1 | -9.1 | -7.9 | -7.4 | -7.5 | -7.3 | -7.3 | -7.2 |
|  | MYC | -7.7 | -7.4 | -7.2 | -7.1 | -7.5 | -7.4 | -7.2 |
|  | PYY | -8.2 | -7.8 | -7.3 | -7.4 | -7 | -6.3 | -6.7 |
|  | SST | -7.6 | -7.4 | -6.6 | -6.5 | -6.1 | -6 | -5.4 |
| HCC | AURKA | -11.7 | -10.3 | -10.1 | -9.7 | -9.1 | -10.8 | -9.3 |
|  | CDC20 | -10.1 | -9.7 | -9.9 | -10.1 | -9.4 | -10.5 | -9.9 |
|  | CCNB2 | -11.2 | -10.2 | -9.8 | -9.9 | -9.3 | -9.3 | -9.7 |
|  | CDK1 | -10.9 | -10.3 | -10.2 | -10.2 | -9.2 | -9.3 | -9.1 |
|  | TOP2A | -10.2 | -9.1 | -9.9 | -9.1 | -9.1 | -8.2 | -8.7 |
|  | PRC1 | -9.7 | -9.3 | -9 | -8.8 | -8.1 | -7.7 | -8.5 |
|  | RRM2 | -8.8 | -8.6 | -8.7 | -8.4 | -8.3 | -8.4 | -9.3 |
|  | CCNB1 | -9.1 | -8.8 | -8.7 | -8.6 | -8 | -8.9 | -8.2 |
|  | RFC4 | -9.1 | -8.4 | -8.3 | -8 | -8.2 | -7.3 | -7.8 |
|  | BUB1 | -9.1 | -8 | -7.8 | -7.8 | -7.8 | -7.3 | -8.1 |
| T2D | PTP1B | -11.1 | -10.9 | -9.9 | -10.6 | -10.1 | -9.1 | -9.8 |
|  | ALB | -10.6 | -9.7 | -10.4 | -8.7 | -9.2 | -9.4 | -9.3 |
|  | LUM | -10 | -9.1 | -8.4 | -9.1 | -8.7 | -8.5 | -8 |
|  | KCNJ1 | -9.4 | -9.1 | -8.3 | -8.6 | -8.6 | -7.7 | -8 |
|  | C3AR1 | -9 | -8.3 | -8.8 | -7.9 | -9 | -8.1 | -8.1 |
|  | HHEX | -9.1 | -8.9 | -8.1 | -9 | -7.9 | -7.5 | -7.8 |
|  | AMPK | -9.2 | -7.7 | -7.9 | -7.6 | -7.8 | -7.9 | -7.9 |
|  | IL6 | -8.4 | -8 | -8.5 | -7.6 | -8 | -8.1 | -7.7 |
|  | ATF3 | -8.6 | -7.9 | -8.8 | -7.8 | -7.4 | -6.8 | -7.8 |
|  | EGF | -7.8 | -7.6 | -7.3 | -7.3 | -6.6 | -7 | -6.6 |
