# Supplementary material for: Exploring common genomic biomarkers to disclose common drugs for the treatment of colorectal cancer and hepatocellular carcinoma with type-2 diabetes through transcriptomics analysis
Source: PLoS One. 2025 Mar 24;20(3):e0319028. doi: 10.1371/journal.pone.0319028 (PMC11932495; doi:10.1371/journal.pone.0319028)
Supplement: S14 Table — (DOCX) [file pone.0319028.s021.docx]

| **S14 Table: 3D visualization of strong binding interactions between target proteins and drugs.** | | | |
| --- | --- | --- | --- |
| **Protein & Ligand complex** | **Binding Affinity (kCal/ mol)** | **The 3D View of**  **Complexes** | **Target-Ligand Interaction highlighting targeted residues** |
| **MMP9**  **&**  **Digitoxin** | -11.5 | 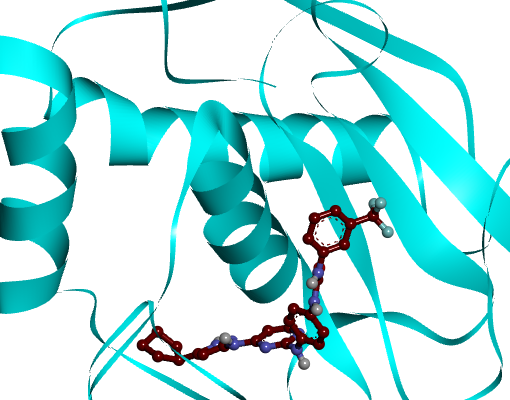 | 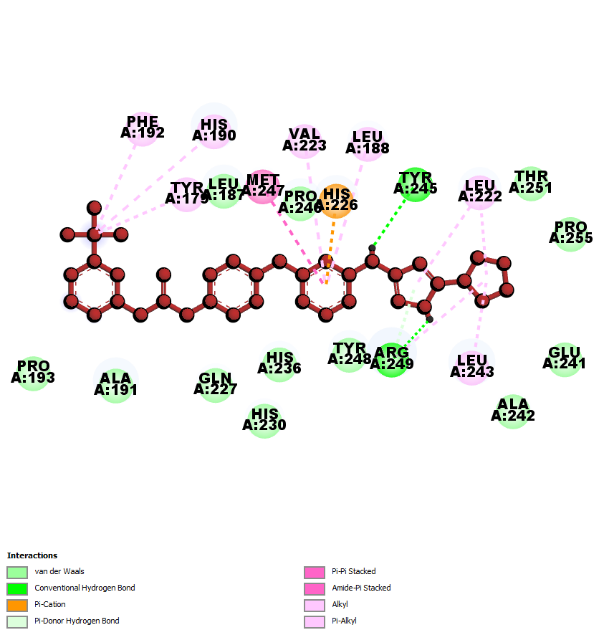 |
| **SPP1**  **&**  **AMG-900** | -10.2 | 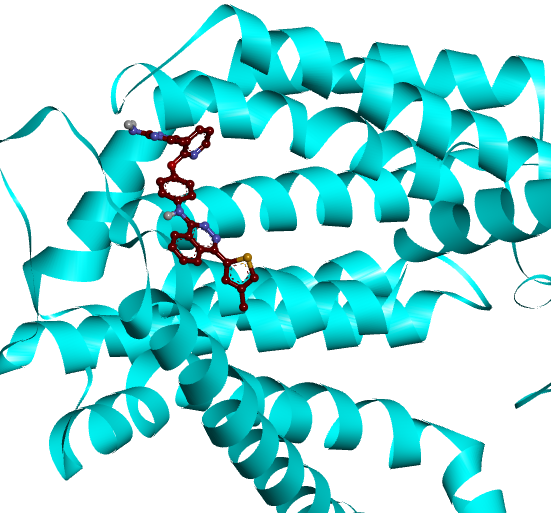 | 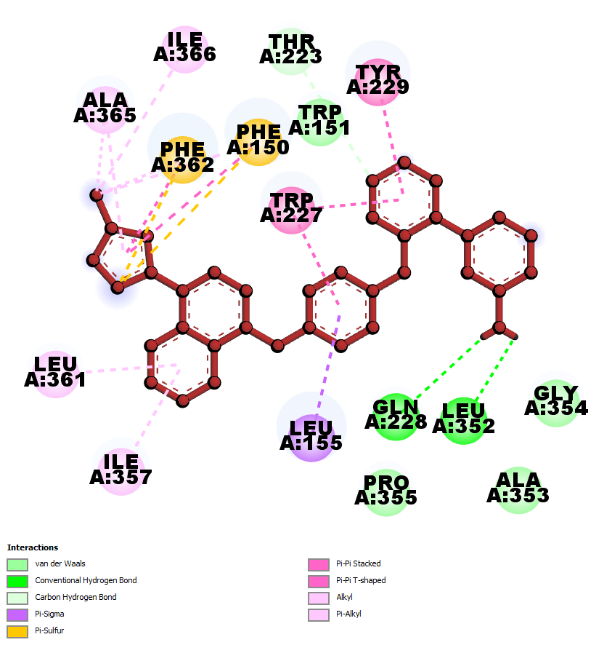 |
| **IL6**  **&**  **Imatinib** | -9.5 | 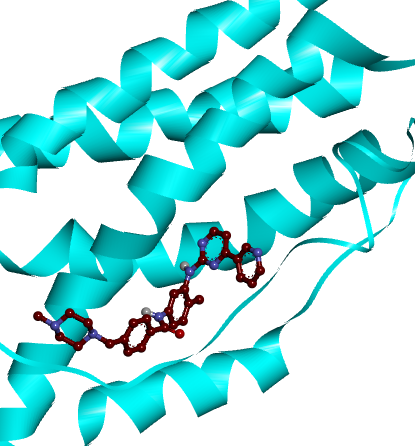 | 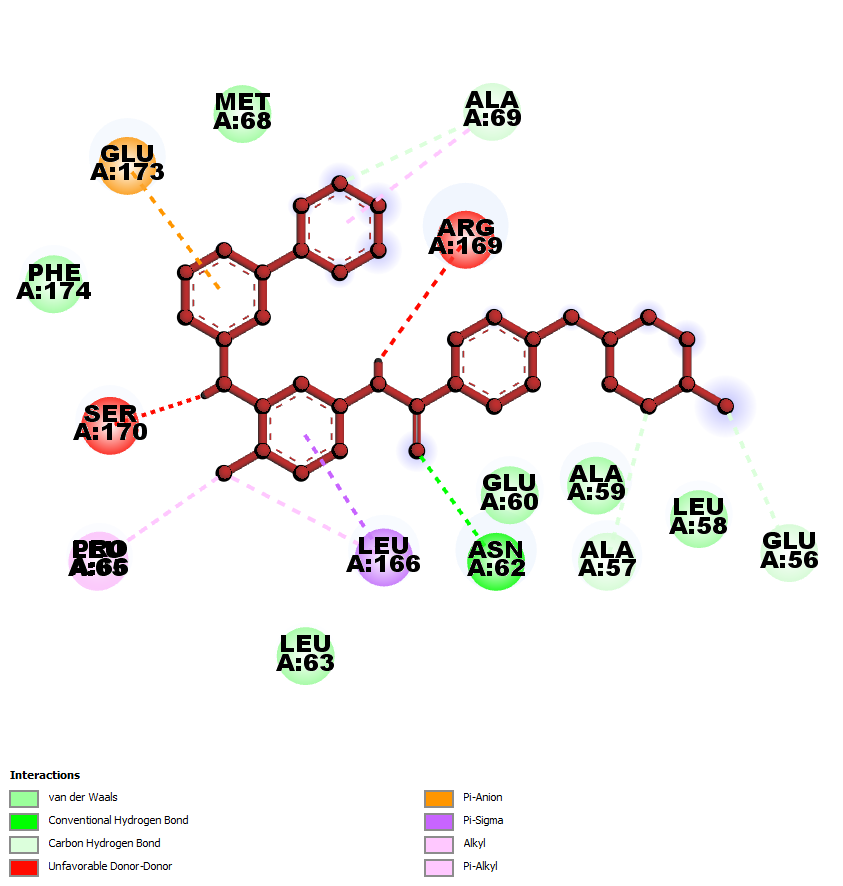 |
