# Supplementary material for: Exploring common genomic biomarkers to disclose common drugs for the treatment of colorectal cancer and hepatocellular carcinoma with type-2 diabetes through transcriptomics analysis
Source: PLoS One. 2025 Mar 24;20(3):e0319028. doi: 10.1371/journal.pone.0319028 (PMC11932495; doi:10.1371/journal.pone.0319028)
Supplement: S15 Table — (DOCX) [file pone.0319028.s022.docx]

| S15 Table: Drug-likeness profiles of the top ranked 7 drugs. | | | | | | | | |
| --- | --- | --- | --- | --- | --- | --- | --- | --- |
| Compounds | Molecular weight | LogP | Water Solubility (ESOL) | GI absorption | Log Kp  (Cm/S) | Lipinski rule | | Synthetic accessibility |
|  |  |  |  |  |  | Follow | Violation |  |
| Digitoxin | 764.95 | 3.247 | -4.41 | Low | -9.65 | 3 | 2 | 8.74 |
| Amg-900 | 503.591 | 4.636 | -2.975 | Low | -5.75 | 4 | 1 | 3.96 |
| Irinotecan | 586.68 | 3.705 | -5.71 | High | -7.22 | 4 | 1 | 5.59 |
| Imatinib | 493.615 | 4.5903 | -5.07 | High | -6.81 | 5 | 0 | 3.78 |
| Midostaurin | 570.64 | 4.05 | 2.892 | High | -6.36 | 4 | 1 | 5.41 |
| Linsitinib | 421.49 | 4.442 | -5.46 | High | -5.91 | 5 | 0 | 4.21 |
| Camptosar | 586.68 | 3.705 | -5.71 | High | -7.22 | 4 | 1 | 5.59 |
