# Supplementary material for: Exploring common genomic biomarkers to disclose common drugs for the treatment of colorectal cancer and hepatocellular carcinoma with type-2 diabetes through transcriptomics analysis
Source: PLoS One. 2025 Mar 24;20(3):e0319028. doi: 10.1371/journal.pone.0319028 (PMC11932495; doi:10.1371/journal.pone.0319028)
Supplement: S16 Table — (DOCX) [file pone.0319028.s023.docx]

| **S16 Table: Frontier molecular orbitals diagram for HOMO and LUMO of proposed drug compounds** | | |
| --- | --- | --- |
| **DRUGS** | **HOMO** | **LUMO** |
| **Digitoxin** | **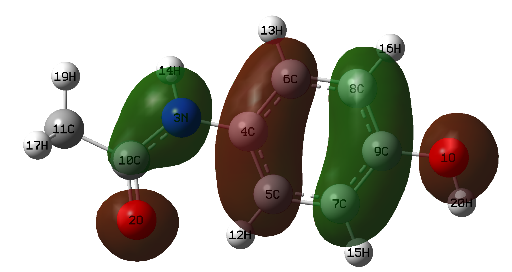** | **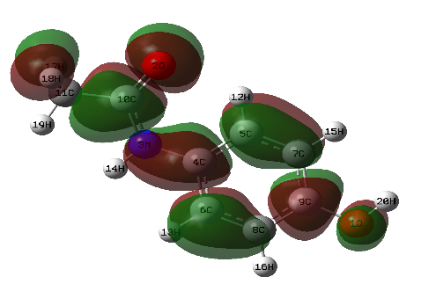** |
| **AMG_900** | **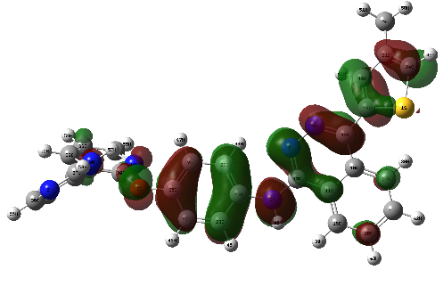** | **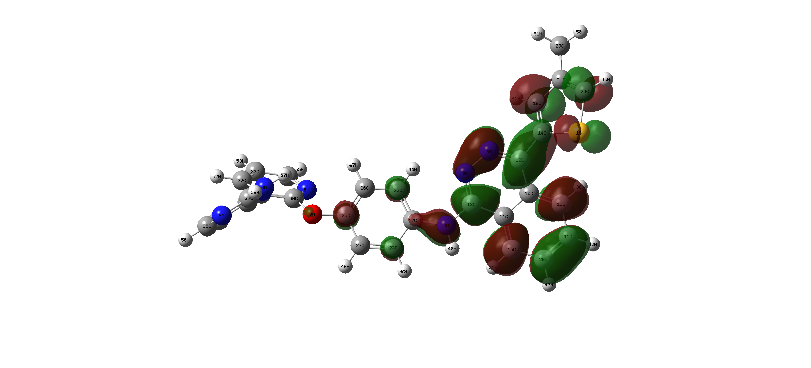** |
| **Imatinib** | **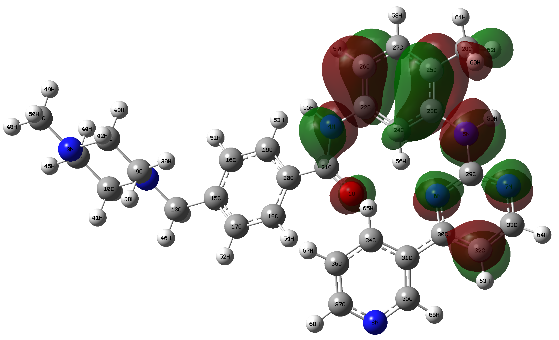** | **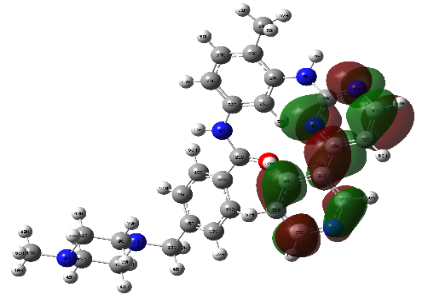** |
| **Irinotecan** | **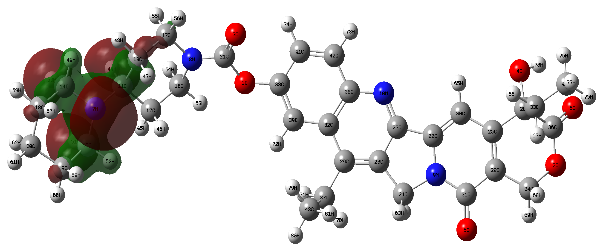** | **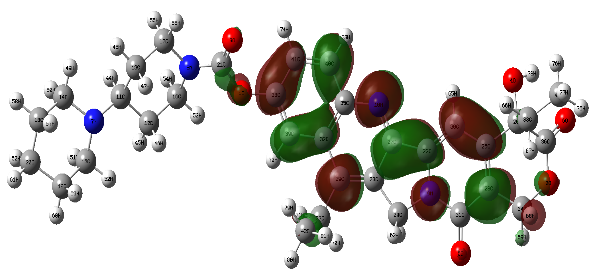** |
| **Linsitinib** | **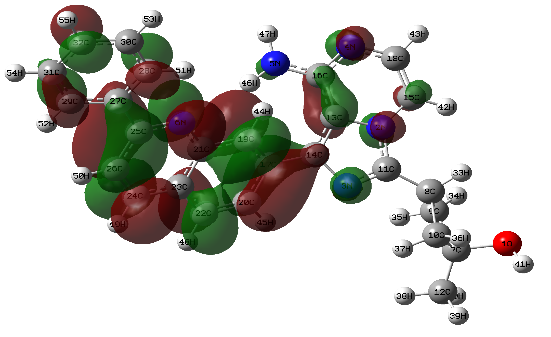** | **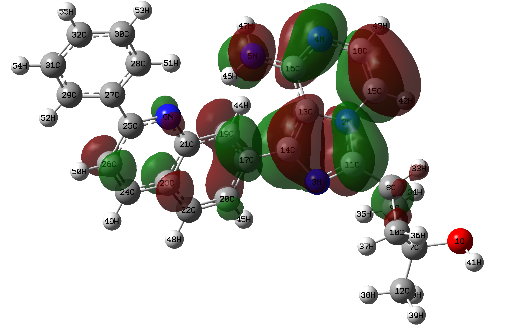** |
| **Midostaurin** | **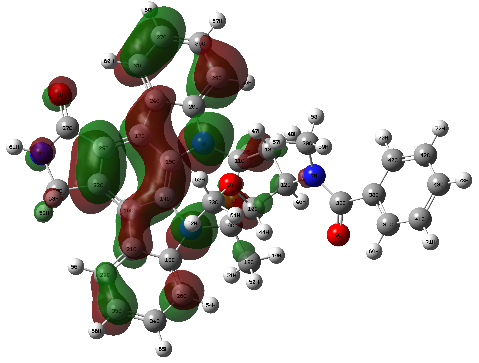** | **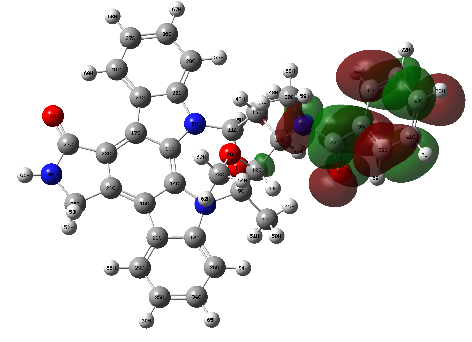** |
| **Camptosar** | **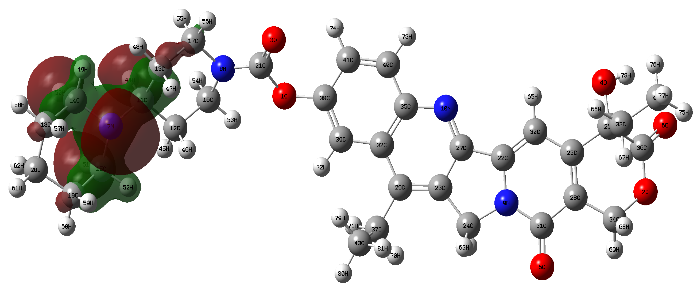** | **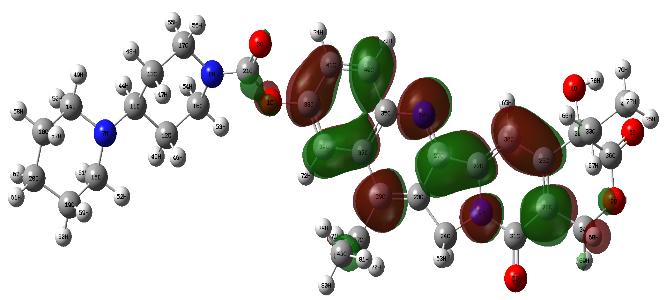** |
